# Constitutive G_s _activation using a single-construct tetracycline-inducible expression system in embryonic stem cells and mice

**DOI:** 10.1186/scrt52

**Published:** 2011-03-04

**Authors:** Edward C Hsiao, Trieu D Nguyen, Jennifer K Ng, Mark J Scott, Wei Chun Chang, Hengameh Zahed, Bruce R Conklin

**Affiliations:** 1 Gladstone Institute of Cardiovascular Disease, 1650 Owens St., San Francisco, CA 94158, USA; 2 Division of Endocrinology and Metabolism, Department of Medicine, 400 Parnassus Ave., University of California, San Francisco, CA 94143-1222, USA; 3 Department of Cellular and Molecular Pharmacology, 600 16th Street Rm. S-222, University of California, San Francisco, CA 94158-2140, USA; 4 Gladstone Institute of Neurological Disease, 1650 Owens St., San Francisco, CA 94158, USA; 5 Biomedical Sciences Graduate Program, 513 Parnassus Ave. Rm. HSE-1285, University of California, San Francisco, CA 94158-0505, USA; 6 Department of Medicine, 505 Parnassus Ave., University of California, San Francisco, CA 94143, USA

## Abstract

**Introduction:**

The controlled expression of many genes, including G-protein coupled receptors (GPCRs), is important for delineating gene functions in complex model systems. Binary systems for inducible regulation of transgene expression are widely used in mice. One system is the tTA/TRE expression system, composed of a tetracycline-dependent DNA binding factor and a separate tetracycline operon. However, the requirement for two separate transgenes (one for each tTA or TRE component) makes this system less amenable to models requiring directed cell targeting, increases the risk of multiple transgene integration sites, and requires extensive screening for appropriately-functioning clones.

**Methods:**

We developed a single, polycistronic tetracycline-inducible expression platform to control the expression of multiple cistrons in mammalian cells. This platform has three basic constructs: regulator, responder, and destination vectors. The modular platform is compatible with both the TetOff (tTA) and TetOn (rtTA) systems. The modular Gateway recombineering-compatible components facilitate rapidly generating vectors to genetically modify mammalian cells. We apply this system to use the elongation factor 1α (*EF1α*) promoter to drive doxycycline-regulated expression of both the fluorescent marker mCherry and an engineered G_s_-coupled GPCR "Rs1" separated by a 2A ribosomal skip site.

**Results:**

We show that our combined expression construct drives expression of both the mCherry and Rs1 transgenes in a doxycycline-dependent manner. We successfully target the expression construct into the Rosa26 locus of mouse embryonic stem (ES) cells. Rs1 expression in mouse ES cells increases cAMP accumulation via both basal and ligand-induced G_s _mechanisms and is associated with increased embryoid body size. Heterozygous mice carrying the Rs1 expression construct showed normal growth and weight, and developed small increases in bone formation that could be observed in the calvaria.

**Conclusions:**

Our results demonstrate the feasibility of a single-vector strategy that combines both the tTA and TRE tetracycline-regulated components for use in cells and mouse models. Although the *EF1α *promoter is useful for driving expression in pluripotent cells, a single copy of the *EF1α *promoter did not drive high levels of mCherry and Rs1 expression in the differentiated tissues of adult mice. These findings indicate that promoter selection is an important factor when developing transgene expression models.

## Introduction

G-protein coupled receptors (GPCRs) are the largest family of cell-surface receptors. GPCRs mediate a wide variety of biological processes and responses to extracellular signals and are the major targets for over 40% of modern pharmaceuticals [1]. However, the diversity of the GPCR family, as well as the presence of constitutive signaling in some GPCRs, poses major challenges for studying the effects of GPCR signaling in *in vitro *and *in vivo *systems.

Receptors activated solely by synthetic ligands (RASSLs) provide one method for experimentally manipulating the timing and activation of G-protein pathways [2,3]. RASSLs are engineered receptors that no longer respond to endogenous hormones, but are activated by synthetic small-molecule drugs. They have proven valuable for studying the roles of activated G-protein signaling in complex systems, including cardiomyocyte function [4], neurological development and function [5-7], and bone development [8-11].

Since many GPCRs show both constitutive and ligand-activated signaling, having temporal and tissue-specific control of GPCR transgene expression is important for delineating specific signaling functions. A variety of binary expression systems are used in genetic model organisms to achieve regulated expression, including the *GAL4*/UAS [12] and the tetracycline-regulated (tTA/TRE) system [13]. The tTA/TRE system uses two separate components to regulate spatial and temporal gene expression: a regulator construct, containing a promoter to drive tissue-specific expression of either the tetracycline-controlled transactivator (tTA; TetOff) or the reverse tetracycline-controlled transactivator (rtTA; TetOn); and a responder construct bearing the minimal TetO promoter and a tetracycline-responsive element (TRE) to temporally control expression of the gene of interest. This system has been shown to control the expression of "Rs1," an engineered G_s_-coupled GPCR with high constitutive G_s _signaling activity, for regulated expression in mouse osteoblasts [8-10].

Although the binary tTA/TRE system is powerful for generating tissue-specific tetracycline-inducible transgenic mouse models, the requirement for two separate constructs poses significant challenges for using the system in cell lines. Various methods are available for introducing the responder and regulator constructs independently (for example, by mixing lentiviral constructs or sequential transgene introduction). These methods have significant limitations: the exact ratios of the responder and regulator plasmids are not fully controlled, the constructs may integrate at multiple sites (thus increasing the risk of off-target effects), and separate drug resistance genes may be needed to ensure that both constructs are maintained as stable integrants.

To address these challenges and determine if global G_s _signaling affects early mouse development, we developed a single-vector polycistronic Tet-inducible expression platform based on a modular construction strategy. This platform uses three vectors that can be recombined to form a single Tet-inducible expression vector. In contrast to several prior methods for combining the tTA/TRE components [14-22], our platform is compatible with both the TetOff (tTA) and TetOn (rtTA) technologies and is rapidly adaptable for multiple gene delivery backbones commonly used in eukaryotic cell lines (for example, lentivirus or Rosa26 targeting).

In this study, we demonstrate the utility of our single-vector constructs in mouse embryonic stem (ES) cells and mice. We show that expression of both mCherry and the G_s_-coupled RASSL Rs1 can be tightly controlled with doxycycline in mouse ES cells. In addition, we show that a Rosa26 knock-in construct using the *EF1α *promoter to drive expression of Rs1 and mCherry is functional in mice, but that the *EF1α *promoter does not drive high expression of the transgenes in all tissues of adult mice. Finally, our results indicate that low-level expression of a GPCR with constitutive G_s _activity leads to a mild increase in calvarial bone formation.

## Materials and methods

### Plasmids

All plasmid constructs used in this study, their Addgene accession numbers, and maps are summarized in Table S1 in Additional File 1 and Figures S1A-H in Additional File 2.

#### attL1L3 entry vectors

The pEntr2B entry vector (Invitrogen, Carlsbad, CA, USA) was digested with PstI and XhoI to remove the *att*L2 site. Oligonucleotides containing SalI-XhoI-Bsu36I-NdeI-PacI-EcoRI-NotI flanking the *att*L3 sequence on the 5' side and PstI on the 3' side were ligated in to create an intermediate plasmid carrying *att*L1 and *att*L3 sites (from pDest R4-R3, Invitrogen). A LoxP sequence was introduced at the XhoI site, and the 500-bp chicken β-globin (CBG) HS4 insulator sequence [23] was introduced at the Bsu36I/NdeI sites. The tTA and pA sequences were subsequently cloned into the EcoRI/NotI sites by PCR cloning from pUHG15-1 [24] to generate pEntL1L3 tTA-2. The *EF1α *promoter from pORF9 (Invivogen, San Diego, CA, USA) was cloned into the PacI site to generate the final entry vector pEntL1L3 EF1α-tTA-2. The construct was verified by sequencing.

#### attR3L2 entry vectors

The pEntr2B entry vector was digested with AflII and XhoI to remove the *att*L1 and ccdb genes. Oligos containing the *att*R3 site flanked by AflII on the 5' end and a polylinker (NotI-PacI-Bsu36I-NdeI-XhoI) on the 3' end were ligated to generate the empty pEntR3L2-MCS intermediate. Using PCR cloning, the TetO-beta globin intron sequence from pTetO (pUHD10.3) [13,24] was cloned into the NotI/PacI sites in the reverse orientation, and a 3' SbfI site was introduced. The pA sequence from pDest27 (Invitrogen) was then cloned into the NotI/SbfI sites, again in reverse orientation. A LoxP site was introduced into the NdeI/XhoI sites, allowing full excision of the targeted construct when used together with the LoxP site in the pEntl1L3 vector. The CBG core insulator was then cloned into the Bsu36I/NdeI sites to generate the starting entry vector pEntR3L2 TetO(fl)-2. The construct was fully sequence verified. The mCherry-P2A-Rs1 RASSL cassette was cloned into the NotI site to generate pEntR3L2 TetO(sh) mCh-Rs1-2. The Rs1 was PCR amplified from pUNIV-SIG-5HTR4D100A [8] and the mCherry was PCR cloned from pRset-mCherry [25]. The self-cleaving 2A site generates two separate peptides in equal concentrations [26,27] via a ribosomal "skip" mechanism just before the C-terminal end of the 2A peptide [28] and has been useful for making multicistronic reporters [29]. During sequencing of the final pEntR3L2 TetO(sh) mCh-Rs1-2 construct (abbreviated pEntR3L2 TetO-mCh-Rs1 in the remainder of this study), the TetO region was noted to carry a deletion of two of the 7 tTA binding site repeats. However, the shortened TetO retained comparable function to the full-length TetO (designated with "fl" for full length) in separate *in vitro *assays (data not shown).

#### Rosa26 R1R2 destination vector (pRosa26 R1R2 RexNeo)

The Rosa26-1 plasmid [30] [Addgene: 21714] was digested at the XbaI cloning site and a PCR product containing the *att*R1-ccdb-*att*R2 sequences from pDest27 and an additional PacI site was introduced. The Rex-Neo resistance cassette from the αMHC-eGFP-Rex-Neo lentivirus plasmid [31] was PCR-cloned into the PacI site to allow for antibiotic selection in eukaryotic cells with G418. The modified regions were verified by sequencing.

#### pcDNA3.2-GWdel CMV destination vector

To create a generic promoter-less destination vector, the pcDNA3.2/V5-DEST (Invitrogen) plasmid was digested with SpeI and SacI, filled in using Klenow fragment, and re-ligated. Sequencing verified that the resulting plasmid has the majority of the CMV promoter removed.

### Generating the combined EF1α-tTA/TetO-mCh-Rs1 expression vectors

The final expression vector for introducing the EF1α-tTA/TetO-mCh-Rs1 construct into mammalian cells was generated by LR multisite recombination with LR Clonase Plus (Invitrogen). In accord with protocols provided by the manufacturer, the *att*L1L3 and *att*R3L2 entry vectors were recombined with *att*R1R2 destination vectors (pRosa26 R1R2 RexNeo or pcDNA3.2-GWdelCMV) to generate the expression vectors Exp-R26(EF1α-tTA/TetO-mCh-Rs1) and Exp-pcDNA3.2(EF1α-tTA/TetO-mCh-Rs1). The recombineering junctions within the final expression vectors were verified by sequencing.

### Generating Rosa26-targeted ES cells R26(EF1α-tTA/TetO-mCh-Rs1)

Feeder-independent mouse ES cells (129/OlaHsd strain, sub-line E14Tg2A.4) were maintained in normal growth medium supplemented with murine leukemia inhibiting factor as described [32]. Exp-R26(EF1α-tTA/TetO-mCh-Rs1) was linearized with AgeI. The DNA was electroporated into 3 × 10^6 ^ES cells using a BioRad Gene Pulser XCell at 800 V, 10 μF, and T_**c **_= 0.3. ES cell cultures were selected in normal growth medium [32] supplemented with 175 ng/ml neomycin (Gibco BRL/Invitrogen, Carlsbad, CA, USA) and 1 ng/ml doxycycline (Sigma Aldrich, St. Louis, MO, USA) for 10 days. Twenty-nine colonies were identified and expanded, and colonies were subsequently genotyped by non-radioactive Southern blot (GE RPN3690 kit with CDP-Star) with a 500-bp probe 5' to the targeting vector and following the manufacturer's directions for labeling, hybridization, and detection. This probe was generated by PCR amplification from a plasmid containing the Rosa26 5' region using forward primer ECH141 (5' TTCGCCCTTTAGGAACAAGA 3') and reverse primer ECH142 (5' TTTTGCCAATTGTTCCTGTG 3') from the Rosa26-5' probe plasmid [30] [Addgene: 21715]. The R26(EF1α-tTA/TetO-mCh-Rs1) ES cells were deposited with the MMRRC [MMRRC: 34358].

### Transiently transfected HEK-293 cells

HEK-293 cells were maintained in standard growth medium with 10% FBS. Plasmids were introduced into cells using Lipofectamine 2000 (Invitrogen), according to manufacturer's protocol. Medium was supplemented with 1 ng/ml doxycycline (a tetracycline analog) (Sigma Aldrich). Expression of the transgenes was characterized by fluorescent microscopy and FACS analysis.

### FACS analysis of transgene expression

R26(EF1α-tTA/TetO-mCh-Rs1) ES cells cultured in the presence or absence of doxycycline were treated with 3 mg/ml collagenase I (Worthington Biochemical Corp., Lakewood, NJ, USA) for 10 to 20 minutes until the cells detached from the culture plate. The cells were collected and passed through a cell strainer before FACS analysis using the BD LSRII (BD Biosciences, San Jose, CA, USA) for mCherry expression. FlowJo (TreeStar, Ashland, OR, USA) software was used to analyze flow cytometry data.

### Analysis of embryoid body size

Suspension embryoid bodies (EBs) were formed by seeding 3 × 106 ES cells into a 10-cm low-attachment dish (Corning, Lowell, MA, USA) and allowing the cells to self-aggregate. Cells were maintained for eight days in differentiation medium containing 20% FBS [33,34]. Supplements included 10 ng/ml doxycycline or 50 ng/ml RS67333, as indicated in the experiment figures. Images of EBs were captured over the entire plate by a Zeiss Axiovert 200 M microscope and Axiovision software. EB size was determined by pixel counting using ImageJ software [35,36] and analyzed on Excel (Microsoft Corp., Seattle, WA, USA).

### cAMP accumulation assay

Rs1-expressing cells were seeded in a poly-d-lysine and laminin-coated 24-well plate at a density of 350,000 cells/well. After 24 hours, the cells were treated with 1 μM drug (serotonin or RS67333) for 10 minutes at 37°C. The treatment solution was aspirated off and the cells were lysed. The cell lysates were assayed for cAMP accumulation using the cAMP HiRange HTRF kit (CisBio US, Bedford, MA, USA).

### Generation of EF1α-tTA/TetO-mCh-Rs1 mice

All transgenic mouse studies were approved by and performed in accordance with the Institutional Animal Care and Use Committee and the Laboratory Animal Research Center at the University of California, San Francisco. Mouse chimeras were generated by the Gladstone Transgenic/Gene Targeting Core facility by injection of E14 ES cells carrying the Exp-R26(EF1α-tTA-mCh-Rs1-TetO(sh)-2) knock-in construct and backcrossed to the C57Bl/6 line. Germline transmission was identified by PCR using the primers ECH103 (5' TCATGGAAATCTCCGAGGCG 3') and ECH162 (5' CGAGGGCTCAGTTGGGCTGTTT 3'; 490-bp product) to detect the wildtype R26 allele and ECH137 (5' ACGTCGACTGAATTGGTTCC 3') and ECH161 (5' CCTCTTCCCCTCGTGATCTGCA 3'; 262 bp product) to detect the recombined allele. Genotyping was performed with the REDExtract-N-Amp Tissue PCR kit (Sigma Aldrich) as directed by the manufacturer. Transgene expression was suppressed by continuous administration of doxycycline-impregnated mouse chow (DoxDiet 200 mg/kg; Bio-Serv, Frenchtown, NJ, USA). Transgene expression was activated by switching the mice to regular mouse chow without doxycycline (LabDiet 5053, PMI Nutrition, St. Louis, MO, USA). Both males and females were analyzed together in our experiments as no sex-dependent differences were observed. The ColI(2.3)-tTA and TetO-Rs1 transgenic mice are as described [8]. The EF1α-tTA/TetO-mCh-Rs1 mice are deposited with the Mutant Mouse Regional Resource Center [MMRRC:034320].

### RNA expression analysis

Gene expression analysis was performed on RNA isolated from the selected tissues or from the right humerus of adult experimental animals, as indicated in the figures. Bones for each experiment were batch processed by crushing (multi-sample Bio-Pulverizer, Research Products International, Prospect, IL, USA). All tissues, including the crushed bone, were homogenized (4.5 mm Tissue Tearor, Research Products International) in RNAStat-60 (Iso-Tex Diagnostics, Friendswood, TX, USA) and total RNA was isolated according to the manufacturer's instructions. cDNA was generated using the SuperScript III First Strand Synthesis kit (Invitrogen) as directed by the manufacturer. Expression was assayed using SybrGreen or Taqman primers for Rs1 and GAPDH as described [8,10]; SybrGreen primers for tTA (Forward: 5' CGCCCAGAAGCTAGGTGTAG 3'; Reverse 5' CCCCTTCTAAAGGGCAAAAG 3'); and SybrGreen primers for mCherry (Forward: 5' CCTGTCCCCTCAGTTCATGT 3'; Reverse: 5' GCTTCAAGTAGTCGGGGATG 3'). Taqman probe sets used for expression analysis for apoptosis, pluripotency, and differentiation markers are as follows: Bad (Mm00432042_m1), Ccnb3 (Mm00805476_m1), Mcl1 (Mm01257352_g1), Oct3/4 (Mm00658129_gH) and Sox2 (Mm00488369_s1), Foxa2 (Mm01976556_s1), Nestin (Mm00450205_m1), Nkx2.5 (Mm00657783_m1), ANF (Mm00431717-m1), MyoD (Mm00440387_m1), and Sox17 (Mm00488363_m1). All samples were assayed in technical triplicates, and expression levels were normalized to GAPDH. All qPCR reactions were run on an Applied Biosystems (Foster City, CA, USA) 7900 HT real-time thermocycler.

### Bone densitometry

Mice identified for dual-energy x-ray absorptiometry (DEXA) to measure whole-body areal bone mineral density (BMD) were anesthetized with inhaled isofluorane (1.5 to 2% in oxygen) and scanned on a GE Lunar Piximus2 (Waukesha, WI, USA) as described [8].

## Results

### Assembly of single-vector poly-cistronic Tet-regulated expression vectors

**Figure 1:**
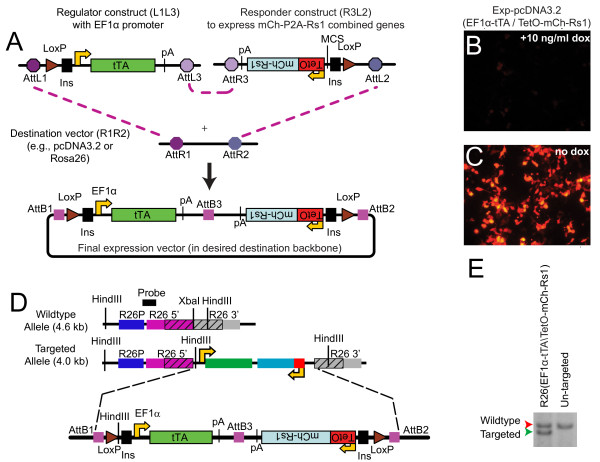
**A single-vector tetracycline construct allows doxycycline-regulated expression**. **(A) **Overview showing the regulator plasmid containing the EF1α-tTA cassette (pEntL1L3-EF1α-tTA, left) and the responder plasmid containing the TetO-mCh-Rs1 cassette (pEntR3L2 TetO-mCh-Rs1, right). The mCherry and Rs1 cistrons are separated by a P2A ribosomal skip sequence to allow simultaneous expression of both peptides. The entry plasmids were recombined using Gateway technology into the desired destination vector containing the AttR1 and AttR2 Gateway sites. The TetO and EF1α-tTA portions are in opposite orientation (indicated by upside-down text) to minimize steric hindrance between the two promoters, as well as potential cross-activation of the TetO by the *EF1α *promoter. In addition, flanking insulator sequences are included to minimize any read-through activation of the constructs by surrounding promoters (such as Rosa26) that may lead to "leakiness" or steric interference from endogenous promoter activity. **(B, C) **HEK-293 cells carrying the Exp-pcDNA3.2(EF1α-tTA/TetO-mCh-Rs1) expression cassette and cultured in doxycycline (suppressed expression) or in the absence of doxycycline (transgene expression allowed) demonstrate doxycycline-dependent mCherry expression. **(D) **Schematic of targeted Rosa26 locus and Southern screening strategy. The Rosa26 locus in E14 ES cells was targeted by homologous recombination with the Exp-R26(EF1α-tTA/TetO-mCh-Rs1) construct. Regions in hatch marks indicate the 5' and 3' homology regions of the targeting vector and the endogenous Rosa26 locus (abbreviated R26 in the figure). The location of the 5' recombination Southern probe and HindIII restriction sites are indicated. **(E) **Southern blots of genomic DNA digested with HindIII and probed as in (D). Heterozygous ES cells at the Rosa26 locus are indicated by the two bands.

To create a single-vector Tet-regulated expression construct, we used a modular cloning strategy employing the Gateway recombineering system [37] with standardized entry vector plasmids to assemble the tetracycline-regulated components into different destination vector backbones (Figure 1A). Briefly, the regulator vector, containing *att*L1 and *att*L3 sites, carries the promoter (that is, the ubiquitous promoter EF1α [38]) used to drive expression of either the tTA or rtTA regulator. The responder vector, containing *att*L2 and *att*R3 sites, carries the minimal TetO tetracycline response element linked to the gene of interest. The destination vector contains the recipient *att*R1 and *att*R2 sites, as well as a selectable marker, and serves as the final backbone for expressing the transgenes in mammalian cells. Multiple Gateway destination vectors based on standard expression plasmids, lentiviral expression vectors, and knock-in constructs are now generally available (e.g., from Invitrogen or Addgene).

We combined the regulator, responder, and destination vectors by Gateway recombineering to create the final single-vector Tet-inducible expression constructs. The regulator and responder cassettes were designed to lie in opposite orientations with the polyA tails nearest each other, thus preventing cross-activation and transcriptional read-through. Additional spacer DNA was placed between the opposing pA sequences to minimize the risk of steric interference of the RNA polymerases. The unidirectional three-way Gateway recombination strategy ensures that the regulator and responder cassettes are always positioned in the proper orientation. In addition, insulator sequences from the chicken β-globin gene flank both sides of the construct to prevent inadvertent read-through into the expression cassettes. Finally, we placed flanking LoxP sites around the construct to allow for Cre-mediated excision of the expression cassette, providing the option of a negative control by excision for epigenetic and integration effects.

### Doxycycline-inducible transgenes in mammalian cells

To test the functionality of our platform, we created the expression construct Exp-pcDNA3.2(EF1α-tTA/TetO-mCh-Rs1) to express the G_s_-coupled RASSL, "Rs1," ubiquitously. The Rs1 and mCherry cistrons are separated by a P2A ribosomal skip sequence, which allows genes to be expressed simultaneously from the same promoter [28]. The 2A sequences have also been used to express peptides in equimolar ratios [26,27]. For ubiquitous tTA transactivator expression, we constructed a regulator vector where the *EF1α *promoter drives tTA. Previous studies indicate that the *EF1α *promoter could direct expression of reporter genes in a variety of cell types and tissues at high levels [38-40]. For the destination vector, we used a modified expression plasmid with the majority of the CMV promoter removed (pcDNA3.2GW-delCMV).

The Exp-pcDNA3.2(EF1α-tTA/TetO-mCh-Rs1) construct was transiently transfected into HEK-293 cells and cultured in either regular medium without doxycycline or in medium with 10 ng/ml doxycycline (Figure 1B,C). Cells cultured in the presence of doxycycline, thus suppressing the transactivator activity, showed no mCherry expression after 24 hours (Figure 1B). In contrast, cells cultured in the absence of doxycycline showed robust mCherry expression (Figure 1C), indicating doxycycline-dependent suppression of transgene expression from our expression construct.

### Targeting the combined mCherry and Rs1 expression construct to the Rosa26 locus

To determine if activation of the G_s _signaling pathway affects embryonic stem cell growth, we created a Gateway-modified destination vector to target the Rosa26 locus (pRosa26 R1R2 RexNeo) for expression of the combined EF1α-tTA and TetO-mCh-Rs1 components. Using Gateway recombineering, we combined the pEntL1L3 EF1α-tTA regulator, pEntR3L2 TetO-mCh-Rs1 responder, and the Rosa26 R1R2 RexNeo destination vectors to generate the Exp-R26(EF1α-tTA/TetO-mCh-Rs1) knock-in expression vector (Figure 1D). Site-specific introduction of the expression cassette into the transcriptionally-permissive Rosa26 locus was performed by gene targeting into E14Tg2A.4 mouse ES cells. Positive clones were identified after G418 selection. Twenty-nine colonies were screened by Southern blot. One ES cell colony (line A6) was identified carrying the correct Rosa26 modification, designated R26(EF1α-tTA/TetO-mCh-Rs1) (Figure 1E), and used for subsequent studies.

### Rs1 activation induces cAMP accumulation in mouse ES cells

**Figure 2:**
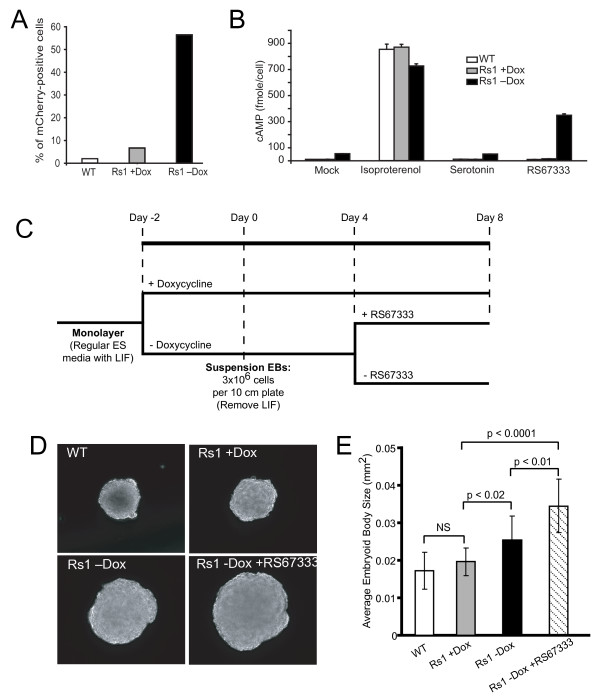
**R26(EF1α-tTA/TetO-mCh-Rs1) function in mouse ES cells**. **(A) **FACS analysis showing doxycycline-inducible mCherry expression in E14 mouse cells carrying the Exp-R26(EF1α-tTA/TetO-mCh-Rs1) construct. **(B) **Induction of Rs1 expression and treatment with the agonist RS67333 results in increased cAMP accumulation in mouse ES cells. Both basal and ligand-induced increases in cAMP are detectable. In addition, serotonin does not induce increased cAMP accumulation in either the wildtype or Rs1-expressing cells. **(C) **Schematic showing the differentiation protocol for making suspension EBs. **(D) **Expression and ligand activation of Rs1 during EB formation results in larger EB size. **(E) **Quantitation of EB size in the different culture conditions using ImageJ. A minimum of 114 EBs were measured for each condition. The analysis was performed on three separate EB differentiation experiments with similar results. Error bars represent average +/- 1 SD.

Analysis of the R26(EF1α-tTA/TetO-mCh-Rs1) mouse ES cell line demonstrated that both the Rs1 and mCherry cistrons were expressed in a doxycycline-dependent manner. ES cells cultured without doxycycline for 48 hours showed brighter labeling with mCherry than ES cells cultured in 10 ng/ml doxycycline. Furthermore, although a low level of mCherry expression was detected when the cells were cultured in doxycycline as compared to unlabeled wildtype ES cells, the percentage of mCherry-positive cells markedly increased when doxycycline was removed (Figure 2A).

Both constitutive and ligand-induced activities of the Rs1 RASSL were preserved in the presence of the P2A ribosomal skip sequence. We previously showed that the Rs1 RASSL is G_s_-coupled and can induce an intracellular increase in cAMP levels by both basal and ligand-induced mechanisms [8,41]. In the R26(EF1α-tTA/TetO-mCh-Rs1) ES cells, cAMP accumulation was minimally increased in mock-treated cells expressing Rs1 as compared to wildtype or doxycycline-suppressed controls (Figure 2B). cAMP levels were further increased in the Rs1-expressing cells cultured with the serotonin receptor agonist RS67333 (Figure 2B) [8,41].

These data show that the R26(EF1α-tTA/TetO-mCh-Rs1) single vector Tet-off polycistronic expression construct in ES cells can be tightly controlled with doxycycline, that both the Rs1 and mCherry cistrons can be expressed from a single locus when separated by a P2A sequence, and that both basal and ligand-mediated increases in cAMP can be induced by the Rs1 RASSL in ES cells.

### Rs1 activation leads to larger embryoid bodies

To determine the effect of higher cAMP levels during the differentiation of ES cells, we cultured the R26(EF1α-tTA/TetO-mCh-Rs1) ES cells for 48 hours either with or without doxycycline and then formed EBs using suspension culture conditions. At Day 4 of differentiation, we divided the EBs cultured without doxycycline into two groups (Figure 2C). One group was treated with the Rs1 agonist RS67333, and the other remained untreated. EB size was analyzed on Day 8. We detected an increase in EB size in both the Rs1-expressing and RS67333-treated groups (Figure 2D, E). R26(EF1α-tTA/TetO-mCh-Rs1) EBs continually cultured with doxycycline to suppress Rs1 expression were the same size as EBs derived from wildtype E14 ES cells. Rs1 mRNA levels were not significantly different between EBs cultured with or without RS67333 (data not shown).

R26(EF1α-tTA/TetO-mCh-Rs1) ES cells expressing Rs1 and grown without doxycycline appeared to proliferate similarly to cells grown with doxycycline. Additionally, the ES cells expressing Rs1 showed no changes in the mRNA levels of apoptosis or proliferation markers (Bad, Mcl1, and Ccnb3) [42] as detected by qPCR. To test whether Rs1 expression may affect cellular differentiation, and thus cell size, we used qPCR to assess the mRNA levels of markers of pluripotency (Oct3/4 and Sox2) and mRNA markers for all three germ layers (Foxa2, Nestin, Nkx2.5, ANF, MyoD, and Sox17) [43]. Rs1-expressing ES cells cultured with or without doxycycline showed no differences in mRNA levels for these genes (data not shown).

These results demonstrate that G_s _signaling during EB formation can increase EB size, but that this increase in size is not a result of detectable changes in cell proliferation, apoptosis, or cellular differentiation.

### EF1α-tTA/TetO-mCh-Rs1 mice

**Figure 3:**
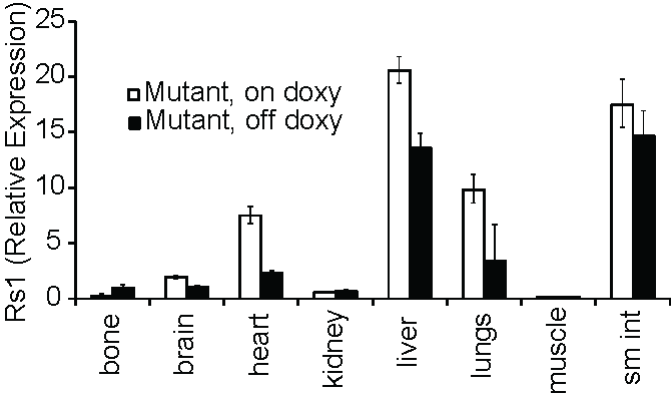
**Rs1 expression in R26(EF1α-tTA/TetO-mCh-Rs1) mice show significant variability and degrees of induction among different tissues**. Rs1 expression was allowed (off doxy) or suppressed (on doxy) in mice for four weeks starting from gestation. mRNA levels were assessed by qPCR for Rs1. Representative mice are shown. Error bars represent average +/- 1 SD of technical triplicates.

Since Rs1 expression in mouse ES cells increased EB size, we sought to determine if global constitutive G_s _signaling could affect tissue development of a whole mouse. ES cells carrying the R26(EF1α-tTA/TetO-mCh-Rs1) construct were injected into mouse blastocysts. Fourteen high-percentage chimeras were identified, and two lines (Line A and Line B) were backcrossed onto the C57Bl/6 background. Mice were maintained and mated on doxycycline-containing chow to suppress transgene expression and minimize any risk of embryonic lethality, as has been hypothesized to occur in diseases with activated G_s _signaling during embryogenesis such as McCune-Albright Syndrome [44]. R26(EF1α-tTA/TetO-mCh-Rs1) mice raised off of doxycycline to allow global expression of Rs1 from gestation were viable and showed no detectable weight, length, or pigmentation changes. Despite the previous reports of ubiquitous *EF1α *promoter activity, expression analysis on four-week-old mice raised on and off doxycycline showed that Rs1 mRNA levels were generally very low and near the detection limit of our qPCR assay. Surprisingly, only whole bone showed an induction of Rs1 expression in mice raised off of doxycycline (Figure 3). In contrast to the induction of Rs1 expression in R26(EF1α-tTA/TetO-mCh-Rs1) mouse ES cells, these findings indicate that the R26(EF1α-tTA/TetO-mCh-Rs1) mice had poor induction of Rs1 expression in most tissues in the absence of doxycycline.

### R26(EF1α-tTA/TetO-mCh-Rs1) test crosses

Since the R26(EF1α-tTA/TetO-mCh-Rs1) mice showed only minimal Rs1 expression *in vivo*, we sought to test whether the tTA regulator portion, the TetO responder portion, or both may be non-functional. Since ColI(2.3)-tTA/TetO-Rs1 mice have a dramatic and easily-detectable bone phenotype [8], we crossed the mice carrying the TetO-Rs1 and R26(EF1α-tTA/TetO-mCh-Rs1) transgenes to test the EF1α-tTA portion and crossed the mice carrying the ColI(2.3)-tTA and R26(EF1α-tTA/TetO-mCh-Rs1) transgenes to test the function of the TetO-mCh-Rs1 portion *in vivo*.

If the EF1α-tTA portion of the R26(EF1α-tTA/TetO-mCh-Rs1) construct was capable of regulating TetO activity *in vivo*, we would predict that the TetO-Rs1 x R26(EF1α-tTA/TetO-mCh-Rs1) mice would show symptoms reminiscent of McCune-Albright syndrome including embryonic lethality, fibrous dysplasia of the bone, short stature, hormonal disturbances, and skin pigmentation defects [45]. However, the TetO-Rs1 x R26(EF1α-tTA/TetO-mCh-Rs1) mice bred and maintained off of doxycycline showed no embryonic lethality (TetO-Rs1 single transgenic = 17; R26(EF1α-tTA/TetO-mCh-Rs1) single transgenic = 16; TetO-Rs1 x R26(EF1α-tTA/TetO-mCh-Rs1) double transgenic = 9; wildtype = 11. Total = 53 mice; Chi-squared *P *= 0.33) and were phenotypically indistinguishable from their littermates.

**Figure 4:**
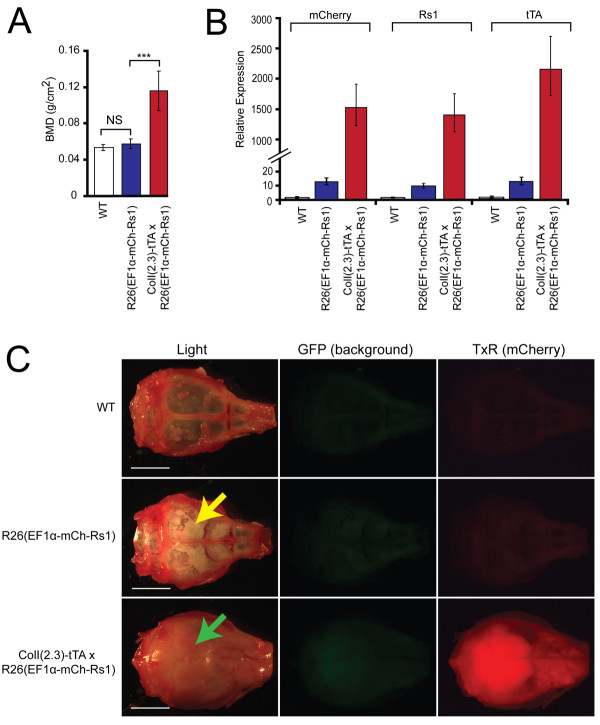
**A single copy of the EF1α-tTA regulator region weakly drives expression of a TetO transgene in mice**. **(A) **Areal bone mineral density by DEXA of nine-week-old mice shows that the ColI(2.3)-tTA x R26(EF1α-tTA/TetO-mCh-Rs1) mice have increased bone mass. *N *= 9 WT, 5 R26(EF1α-tTA/TetO-mCh-Rs1), and 9 ColI(2.3)-tTA x R26(EF1α-tTA/TetO-mCh-Rs1) mice. ***, *P *< 0.0001 vs. wildtype (WT). **(B) **RNA expression levels of Rs1, tTA, and mCherry in the humeri of nine-week-old ColI(2.3)-tTA x R26(EF1α-tTA/TetO-mCh-Rs1) and littermate controls, showing highest expression in the double-mutant mice. Error bars represent means of technical qPCR triplicates +/- 1 SD. **(C) **Fluorescence images of calviaria from representative 16-week-old littermate mice showing strongest mCherry expression [assessed in the Texas Red (TxR) channel] in ColI(2.3)-tTA x R26(EF1α-tTA/TetO-mCh-Rs1) but not wildtype littermates. Although mCherry was not clearly detected in the R26(EF1α-tTA/TetO-mCh-Rs1) calvaria, subtle increases in the mineralization of the skull bones (white patches, as indicated by the yellow arrow) are present indicating functional responses to Rs1. The level of bone formation is lower than that seen in the ColI(2.3)-tTA x R26(EF1α-tTA/TetO-mCh-Rs1) (green arrow), where additional activation of the Rs1 transgene is achieved by adding osteoblast-specific expression of the tTA element.

To determine if the TetO-mCh-Rs1 responder portion of the construct containing a P2A ribosomal skip sequence was functional, we generated ColI(2.3)-tTA x R26(EF1α-tTA/TetO-mCh-Rs1) mice. At nine weeks of age, the double mutants showed a significant increase in bone mineral density (Figure 4A) that was comparable to our published results in the ColI(2.3)-tTA/TetO-Rs1 mice [8]. Further analysis by qPCR of RNA isolated from whole femurs showed that Rs1, mCherry, and tTA expression could be easily detected in the double mutant mice and at relatively comparable levels, but Rs1, mCherry, and tTA expression were not readily detectable in the bones of R26(EF1α-tTA/TetO-mCh-Rs1) single mutant mice (Figure 4B). Images of calvaria from the double mutant mice ColI(2.3)-tTA x R26(EF1α-tTA/TetO-mCh-Rs1) show that red fluorescence from the mCherry could be visualized, but not in the calvaria of the R26(EF1α-tTA/TetO-mCh-Rs1) single mutant or control mice (Figure 4C). However, a small amount of increased bone formation could be detected in the calvaria of R26(EF1α-tTA/TetO-mCh-Rs1) mice as indicated by the increased opacity in the frontal and parietal bones (Figure 4C), consistent with the low level of Rs1 expression previously observed (Figure 3).

These findings show that a single copy of the R26(EF1α-tTA/TetO-mCh-Rs1) construct can drive a low level of Rs1 expression *in vivo *in some tissues, and that the low level of Rs1 expression is due to low activity of the EF1α-tTA portion of the combined transgene. Our results also show that the Rs1 and mCherry cistrons can be expressed from a single locus when separated by a P2A sequence, and that the Rs1 receptor expressed in this manner retains biological activity *in vivo*.

## Discussion

We created a modular system that uses Gateway recombineering technology to combine the binarytetracycline-regulated expression components into a single vector. By using a targeting vector with a known genomic insertion site (Rosa26), we could introduce all of the components needed for tetracycline-regulated expression into a well-characterized and transcriptionally-active locus. This strategy allows us to use the same cell line for both the experimental (that is, Rs1 expressed) and control (that is, Rs1 expression suppressed) conditions, thus significantly decreasing the problem of clonal variability.

Although a variety of methods for combining the tetracycline-inducible expression components into a single vector have been described [14-22,46,47], our modular system has the added advantage of facilitating the rapid interchange of the expression components for tetracycline-induced expression as well as the choice of viral and non-viral delivery backbones. We easily inserted the expression cassettes into two final destination vectors, including a new Gateway-enabled construct for targeting into the mouse Rosa26 locus. We then were able to integrate the combined tTA-TetO construct into the Rosa26 locus of pluripotent mouse cells in a single targeting step.

Our expression strategy also took advantage of a P2A ribosomal skip site to co-express the engineered GPCR Rs1 and the fluorescent marker mCherry. The P2A sequence has been used in a variety of systems to generate independent polypeptides from a single RNA transcript [27-29,48]. In our experiments, combining the mCherry and Rs1 cistrons together allowed us to visualize the expression of mCherry and Rs1 and assess the effect of Rs1-induced G_s _signaling in both ES cell differentiation and mice. Our results indicate that the 2A strategy produces functional membrane-bound receptors and reporter proteins from a single RNA transcript. This strategy will be useful for expressing other GPCRs since it does not require the addition of a large fusion protein domain to the receptor.

In our study, expression of Rs1 and activation of G_s _signaling in mouse ES cells induced larger EB size. Although G_s_-induced cellular proliferation by cholera toxin can increase EB size [49], our results indicated no detectable increase in cellular proliferation, decrease in apoptosis, or change in ES cell differentiation. We were unable to definitively assess whether a change in cell size could be contributing to the increased EB size; however, the difference in observed ES cell proliferation may be a result of lower G_s _activation from the weak *EF1α *promoter activity in our system, lower expression of Rs1 in the ES-cell derived differentiated tissues (as indicated by our mouse results), or differences in how cholera toxin or GPCRs activate the G_s _or non-cannonical GPCR signaling pathways.

Although a single copy of the *EF1α *promoter could drive doxycycline-dependent expression of our construct in ES cells, it was insufficient to drive high expression of the transgenes in differentiated mouse tissues. Our study examined a limited number of founder mice and did not assess whether EF1α-tTA expression varies between founder lines. However, several other reasons could result in low levels of tTA expression: the *EF1α *promoter may not function as robustly in differentiated tissues as in pluripotent cells; the flanking insulator sequences could result in transgene silencing; or the reduced number of TetO repeats in our constructs results in a lower sensitivity to tTA activation. The latter two possibilities are less likely since our results from the mouse crosses indicate that the TetO-mCh-Rs1 portion of the transgene can respond to a separate transgene expressing tTA from the ColI(2.3) promoter, suggesting that the R26(EF1α-tTA/TetO-mCh-Rs1)-targeted locus is not silenced. In addition, prior studies show that constructs with as few as two TetO repeats can be induced by tTA [50]. Finally, while we cannot exclude that the parallel positioning of the EF1α-tTA construct with the endogenous Rosa26 promoter may cause transcriptional interference as previously reported for the CMV promoter [51], this should have been minimized by the use of a 5' insulator sequence.

The observed low levels of Rs1 expression in mouse tissues likely accounts for the absence of embryonic lethality and other pathology we would have expected if our model mimicked McCune-Albright syndrome [44]. In addition, the differential activity of the *EF1α *promoter, or different susceptibility of certain tissues to G_s _signaling, may be contributing to the heterogeneity of the Rs1 expression we observed in our R26(EF1α-tTA/TetO-mCh-Rs1) mice. Increasing the expression of the tTA transactivator by using an alternative ubiquitous promoter, such as the CMV-β actin (CAG) promoter [52], may allow more robust expression of Rs1 and increased activation of the G_s _signaling pathway.

We believe that the modularly-designed single-vector tet system presented here provides an ideal system for the tissue specific expression of tTA or rtTA as well as the controlled expression of a transgene from the tetracycline response element. This system could be used for a variety of genetic studies where a single cassette is advantageous. Having both components of the tet-inducible system in the same cassette could facilitate the genetic modification of ES and induced pluripotent stem cells for regulatory studies as well as for making engineered tissues.

To facilitate these applications, we generated a series of improved vectors (Table S2 in Additional File 3 and Figures S2A-F in Additional File 4). We created a new Rosa26 targeting vector that contains the yeast PI-SceI homing sequence (pR26 R1R2 RexNeo PI-SceI) to simplify linearization of large constructs. We also created pEntL1L3 and pEntR3L2 plasmids without the insulator sequences and adapter plasmids containing only a multiple cloning sequence (pEntL1L3-MCS and pEntR3L2-MCS) to allow use in lentiviral expression constructs (that is, to generate independent tet-regulator and -responder expression constructs in a Gateway-compatible lentivirus backbone such as pLenti6 (Invitrogen)). We believe that these constructs will help advance the generation of new inducible expression models. In addition, these constructs are compatible with new technologies such as transposon-mediated gene transfer, which can move large segments of DNA (> 20 kb) as a single cassette and can be easily mobilized into or excised from the genome [53], as well as newer versions of the tet regulatory components such as Ptet [54].

## Conclusions

The modular system described here allows for rapid generation of tetracycline-regulated expression constructs for gene expression studies in tissue culture, ES cells, and mice. The modular design allows rapid introduction of different combinations of promoters and expressed transgenes. The 2A site can also be used to combine a reporter with an effector gene into a single cistron and is functional even for membrane-bound receptors, such as GPCRs. Finally, a single copy of the *EF1α *promoter was sufficient to induce transgene expression in mouse ES cells but did not result in high expression in the differentiated tissues of a whole mouse. These findings indicate that the specific choice of the promoter is an important consideration for driving high expression of the transgenes and that promoters validated for use in tissue culture models may have different functional characteristics when used *in vivo*. We believe that our modular system for introducing the tetracycline-inducible expression components will facilitate further development of new types of regulated expression constructs for use in a wide variety of cell types, including human ES and induced pluripotent stem cells.

## Abbreviations

EB: embryoid body; *EF1α*: elongation factor 1 alpha; ES: cell, embryonic stem cell; GPCR: G-protein coupled receptor; iPS: cell, induced pluripotent stem cell; RASSL: receptor activated solely by synthetic ligands; Tet: tetracycline; Doxy, doxycycline; TRE: Tet-Responsive Element.

## Competing interests

The authors declare that they have no competing interests.

## Authors' contributions

ECH and BRC conceived of the expression system. ECH designed the components, created expression constructs, generated the mice, and analyzed the progeny. JKN, TDN, MS, and WC created the DNA constructs. JKN, HZ, and TDN analyzed the embryonic stem cell lines. ECH, JKN, TDN, and BRC wrote the paper. All authors read and approved the manuscript.

## Supplementary Material

Additional file 1 **Table S1**. List of plasmids used in this study with brief descriptions and accession numbers. Click here for file

Additional file 2 **Figures S1A-H**. Maps of plasmids used in this study. S1A pEntL1L3 tTA-2.pdf. S1B pEntL1L3 EF1a-tTA-2.pdf. S1C pEntR3L2 TetO(fl)-2 (insulator).pdf. S1D pEntR3L2 TetO(sh) mCh-Rs1-2.pdf. S1E pR26 R1R2 RexNeo PI-SceI.pdf. S1F Exp-R26(EF1a-tTA TetO-mCh-Rs1).pdf. S1G Exp-pcDNA3.2(EF1a-tTA TetO-mCh-Rs1).pdf. S1H pcDNA3.2GW-delCMV.pdf. Click here for file

Additional file 3 **Table S2**. Additional plasmids created as part of this study with brief descriptions and accession numbers. Click here for file

Additional file 4 **Figures S2A-F**. Maps of additional plasmids described in this study. S2A pEntL1L3 MCS.pdf. S2B pEntL1L3 tTA-3 (no ins).pdf. S2C pEntL1L3 rtTA-3 (no ins).pdf. S2D pEntL1L3 EF1a-tTA-3 (no insulator).pdf. S2E pEntR3L2 MCS.pdf. S2F pEntR3L2 TetO(fl)-3 (no insulator).pdf. Click here for file
