# Supplementary material for: Constitutive Gs activation using a single-construct tetracycline-inducible expression system in embryonic stem cells and mice
Source: Stem Cell Res Ther. 2011 Mar 4;2(2):11. doi: 10.1186/scrt52 (PMC3226282; doi:10.1186/scrt52)
Supplement: Additional file 1 — Table S1. List of plasmids used in this study with brief descriptions and accession numbers. [file scrt52-S1.DOC]

**Supplemental Table 1: Plasmid constructs.**

Maps and Addgene deposit numbers are indicated.

| **Map** | **Plasmid** | **Comments** | **Addgene #** |
| --- | --- | --- | --- |
| **L1L3 Entry Vectors** | | | |
| 1A | pEntL1L3 tTA-2 | Contains Gateway L1L3 sites. PacI and Sbf1 can be used to insert promoter of interest to drive tTA expression. Contains insulator sequence. KanR. | 24414 |
| 1B | pEntL1L3 EF1a-tTA-2 | Contains Gateway L1L3 sites and EF1α promoter driving tTA. Contains insulator sequence. KanR | 26803 |
| **R3L2 Entry Vectors** | | | |
| 1C | pEntR3L2 TetO(fl)-2 | Contains Gateway R3L2 sites. Full length (7 repeat) TetO. SbfI, FseI, AvrII, and SalI sites can be used to insert gene of interest, driven by TetO. Contains insulator sequence. KanR. | 24416 |
| 1D | pEntR3L2 TetO-mCh-Rs1  (also referred to as  pEntR3L2 TetO(sh) mCh-Rs1-2) | Contains Gateway R3L2 sites. TetO is missing 2 reapeats. Drives expression a mCherry-P2A-Rs1 cassette. Contains insulator sequence. KanR. | 24417 |
| **Rosa26 Destination Vector** | | | |
| 1E | pR26 R1R2 RexNeo PI-SceI | Murine Rosa26 targeting vector with R1R2 Gateway sites. Includes a ccdB cassette, Rex-Neo selection marker cassette, and DTA selection cassette. AmpR. Deposited in Addgene as “pRosa26 R1-ccdB-R2 RexNeo PI-SceI.” | 24418 |
| **Expression vectors** | | | |
| 1F | Exp-R26(EF1α-tTA/TetO-mCh-Rs1) | Rosa26 targeting vector containing the EF1α-tTA/TetO-mCh-Rs1 cassette flanked by insulator sequences. AmpR. Deposited in Addgene as “Exp-Rosa-EF1a-tTA-mCh-Rs1-TetO(sh)-2” | 24419 |
| 1G | Exp-pcDNA3.2delCMV(EF1α-tTA/TetO-mCh-Rs1) | pcDNA3.2 expression vector with the majority of the CMV promoter deleted, and carrying the EF1α-tTA/TetO-mCh-Rs1 cassette flanked by insulator sequences. AmpR | 24419 |
| **Other Destination Vector** | | | |
| 1H | pcDNA3.2 GW delCMV | pcDNA3.2 vector with majority of the CMV promoter deleted. AmpR | 29496 |
