# Supplementary material for: Constitutive Gs activation using a single-construct tetracycline-inducible expression system in embryonic stem cells and mice
Source: Stem Cell Res Ther. 2011 Mar 4;2(2):11. doi: 10.1186/scrt52 (PMC3226282; doi:10.1186/scrt52)

Supplemental Figure S1A

pEntL1L3 tTA-2

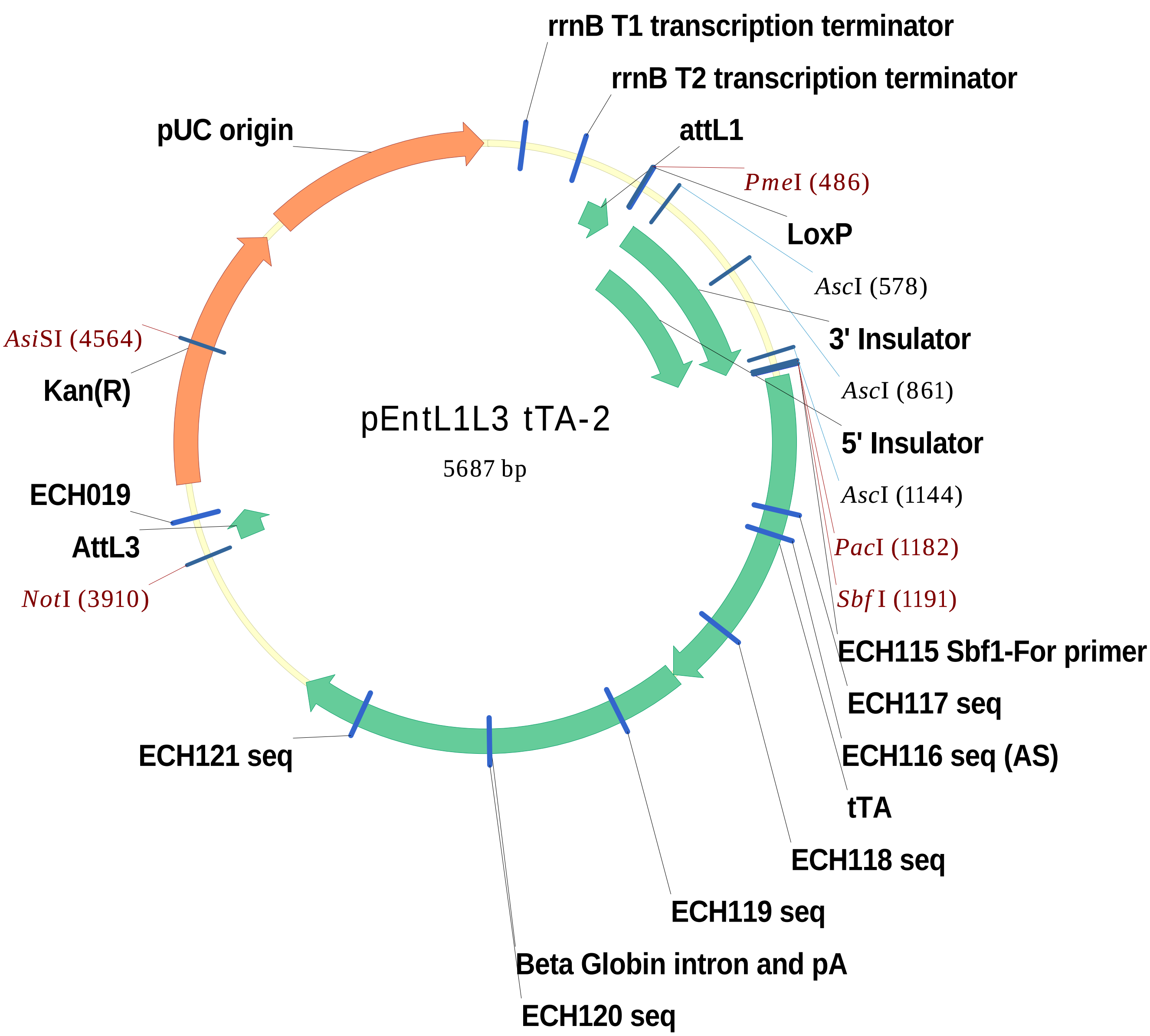

Supplemental Figure S1B

pEntL1L3 EF1a-tTA-2

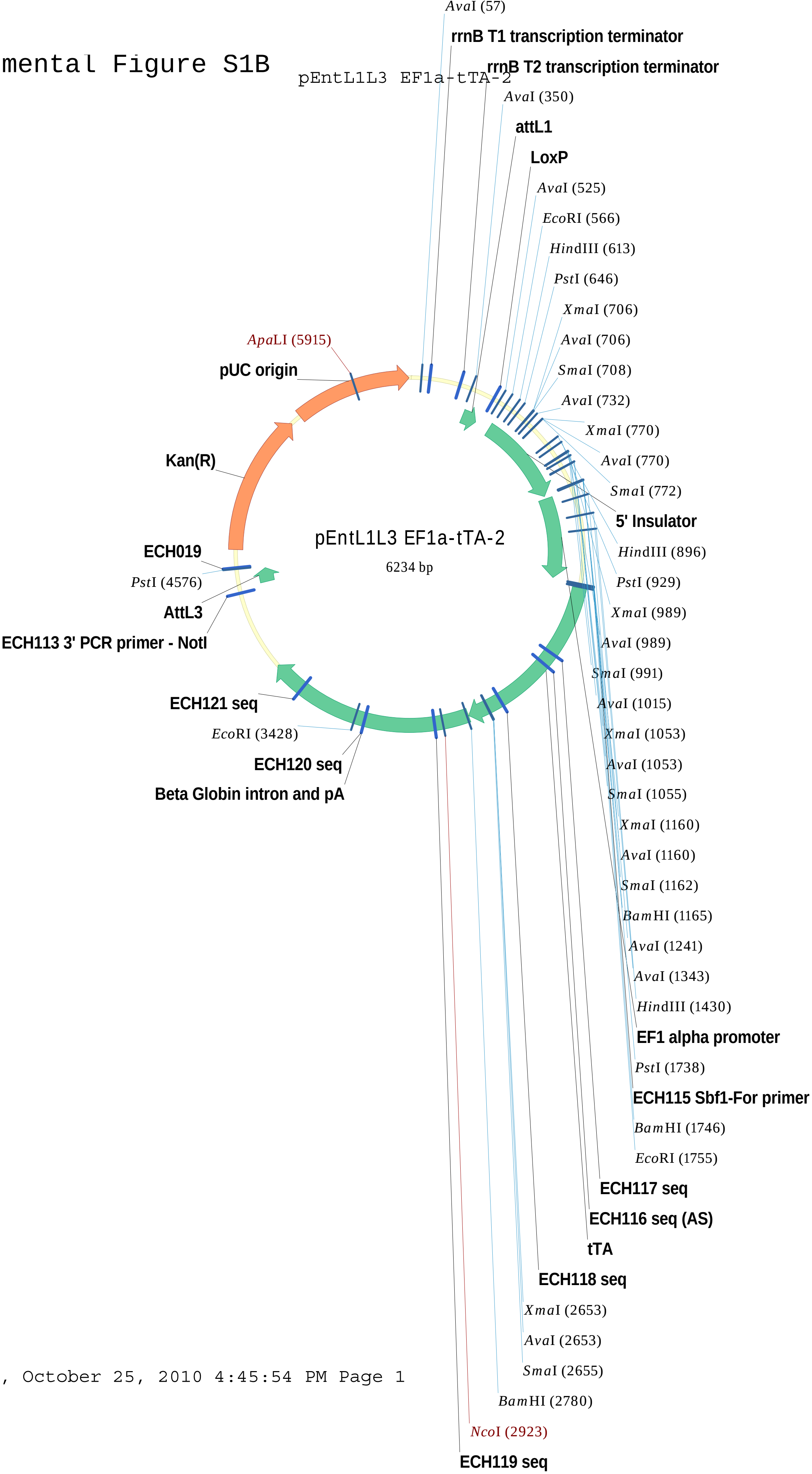

Monday, October 25, 2010 4:45:54 PM Page 1

Supplemental Figure S1C

pEntR3L2 TetO(fl)-2 (insulator)

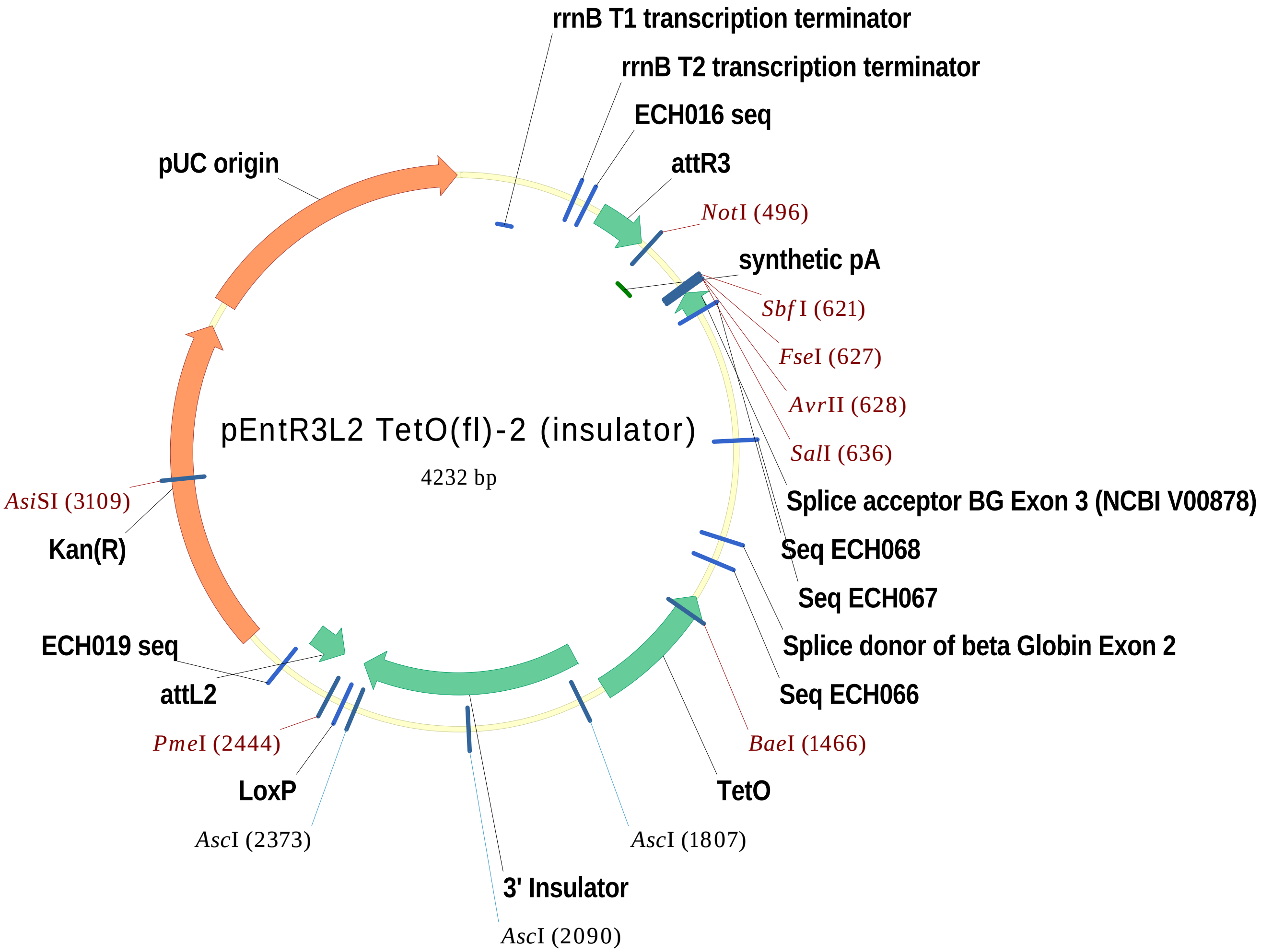

Supplemental Figure S1D

pEntR3L2 TetO(sh) mCh-Rs1-2

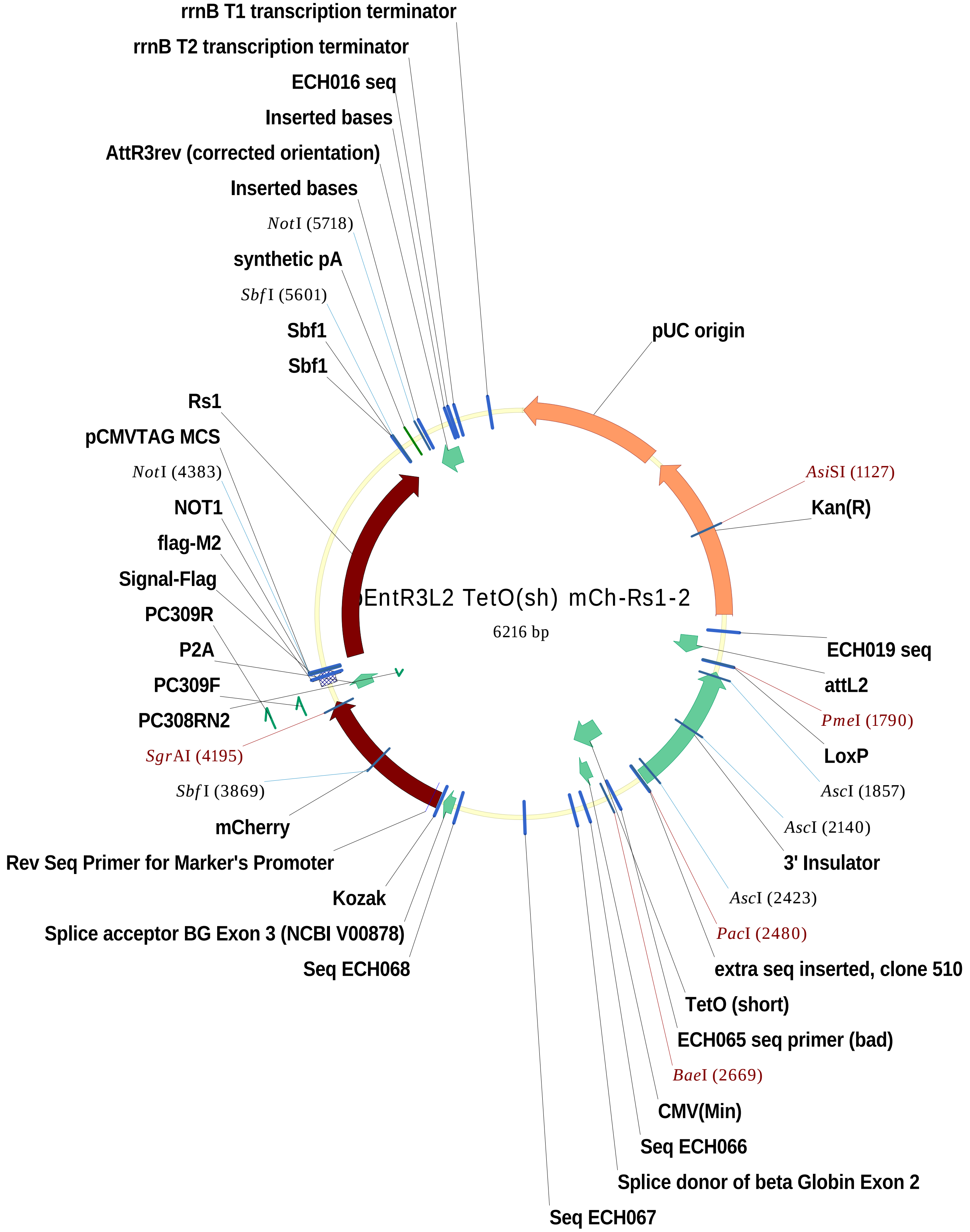

Supplemental Figure S1E

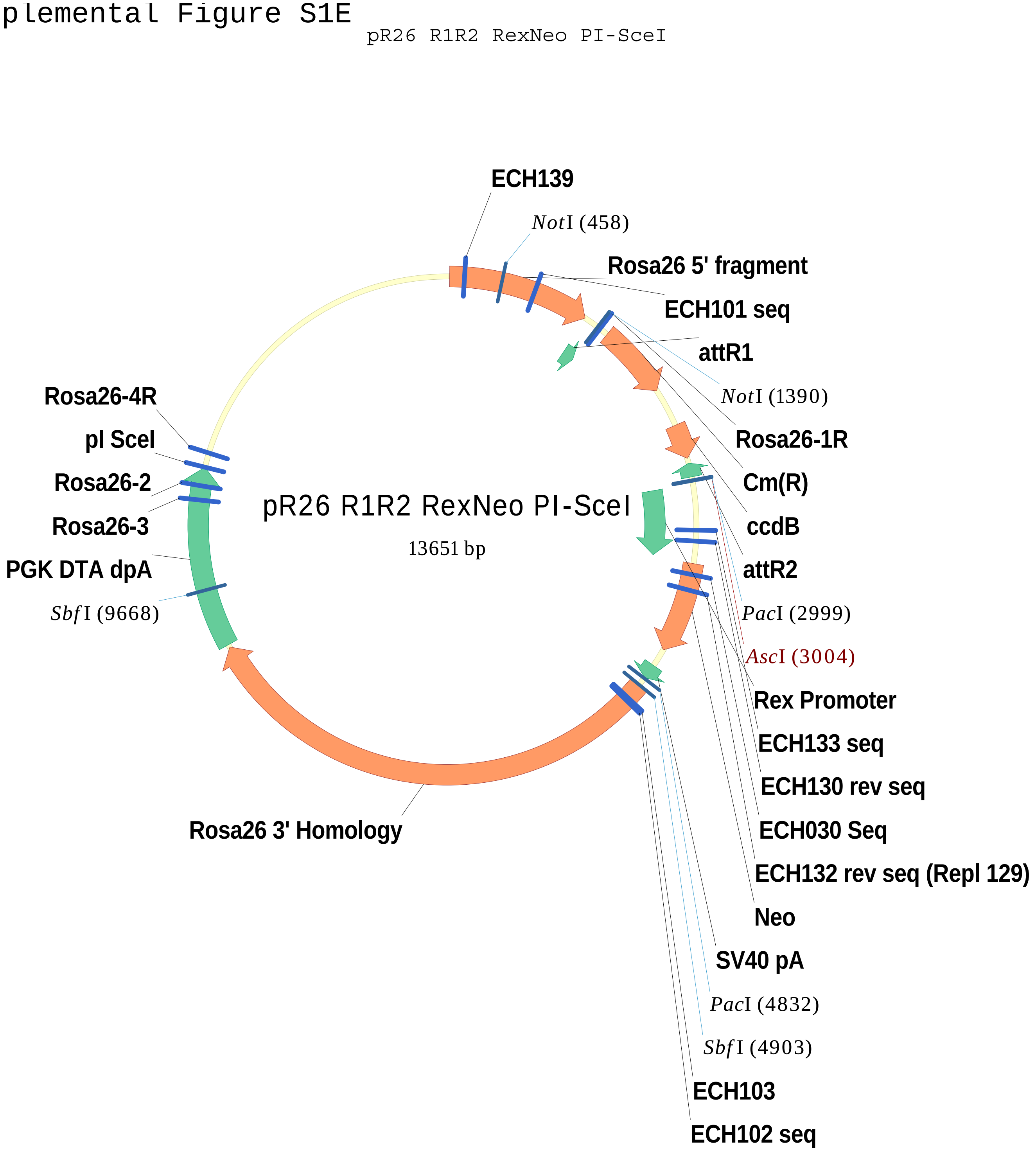

Supplemental Figure S1F

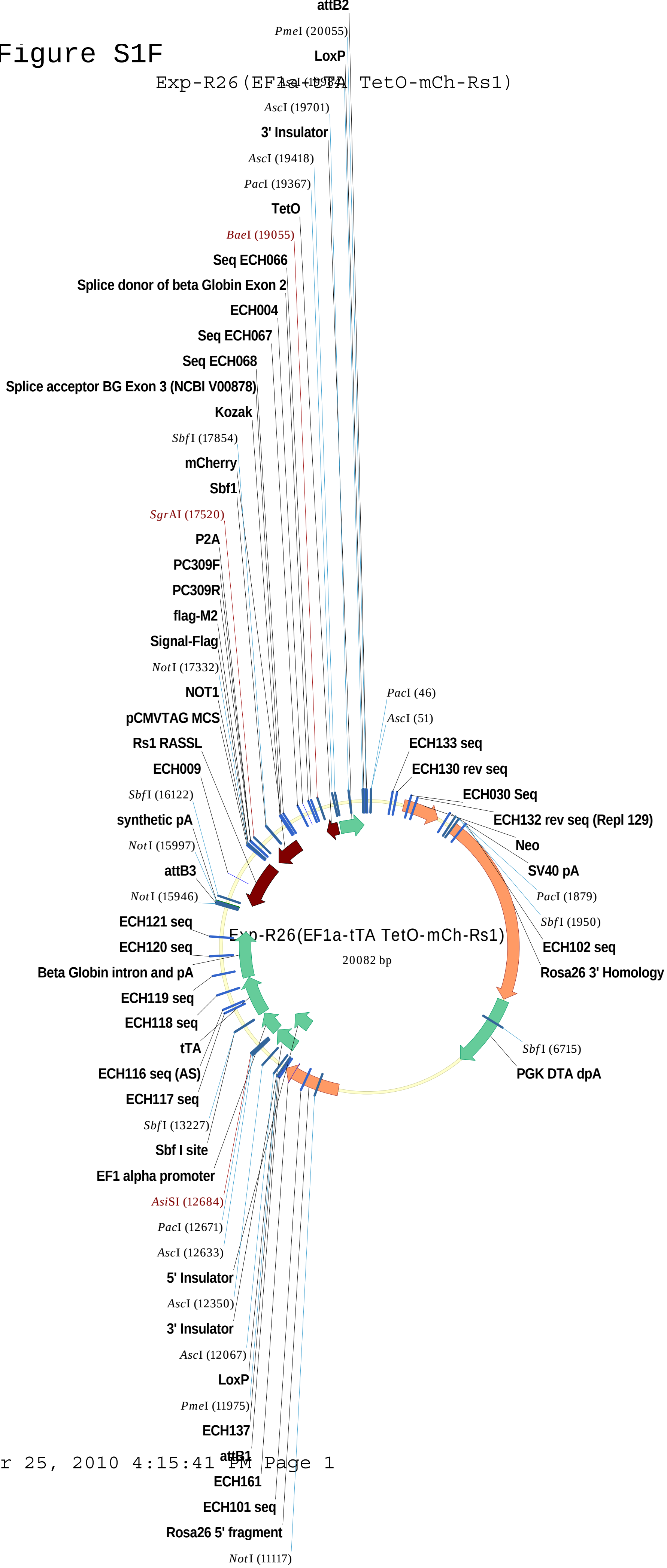

Supplemental Figure S1G

Exp-pcDNA3.2(EF1a-tTA/TetO-mCh-Rs1)

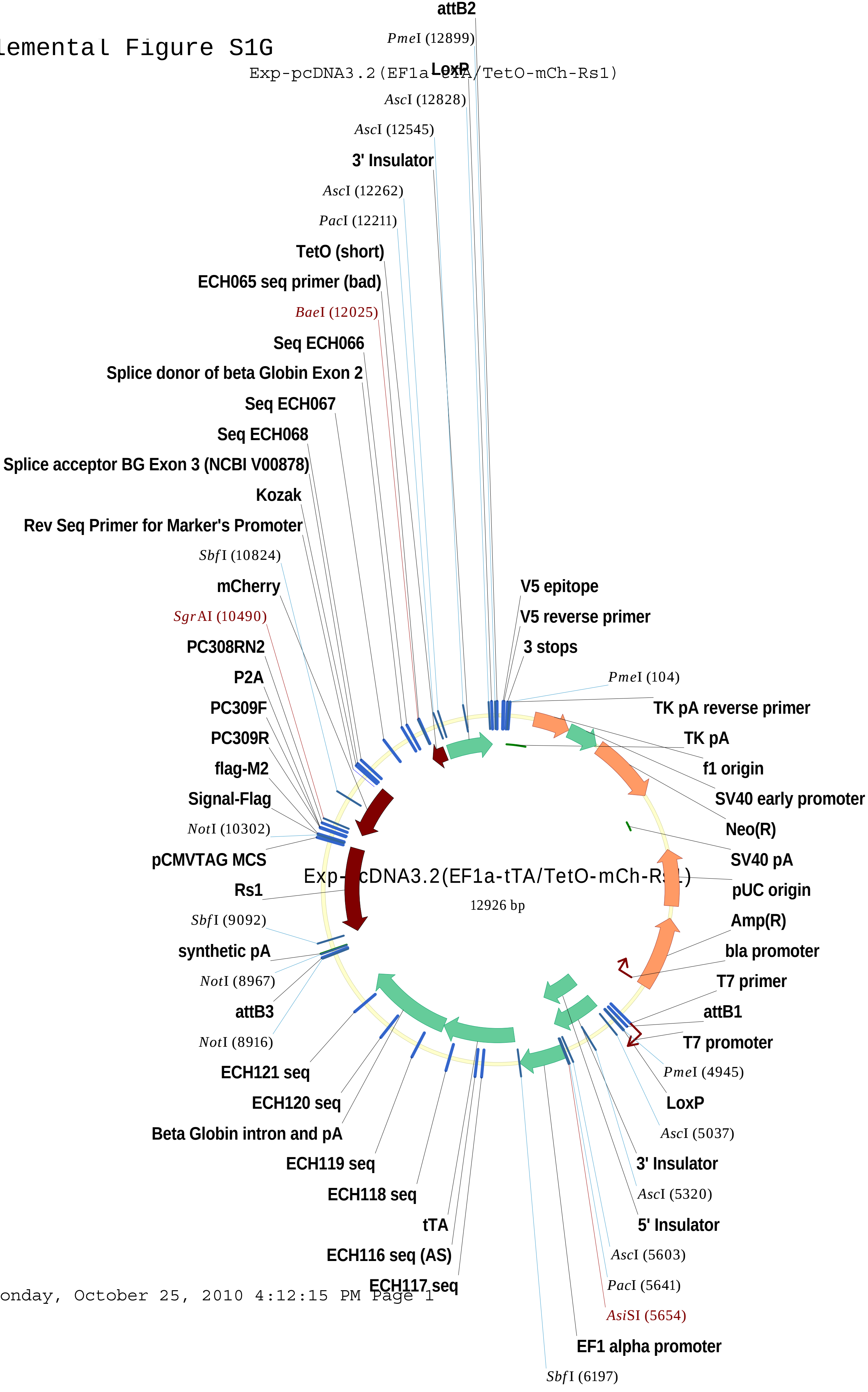

Supplemental Figure S1H

pcDNA3.2 GW del-CMV

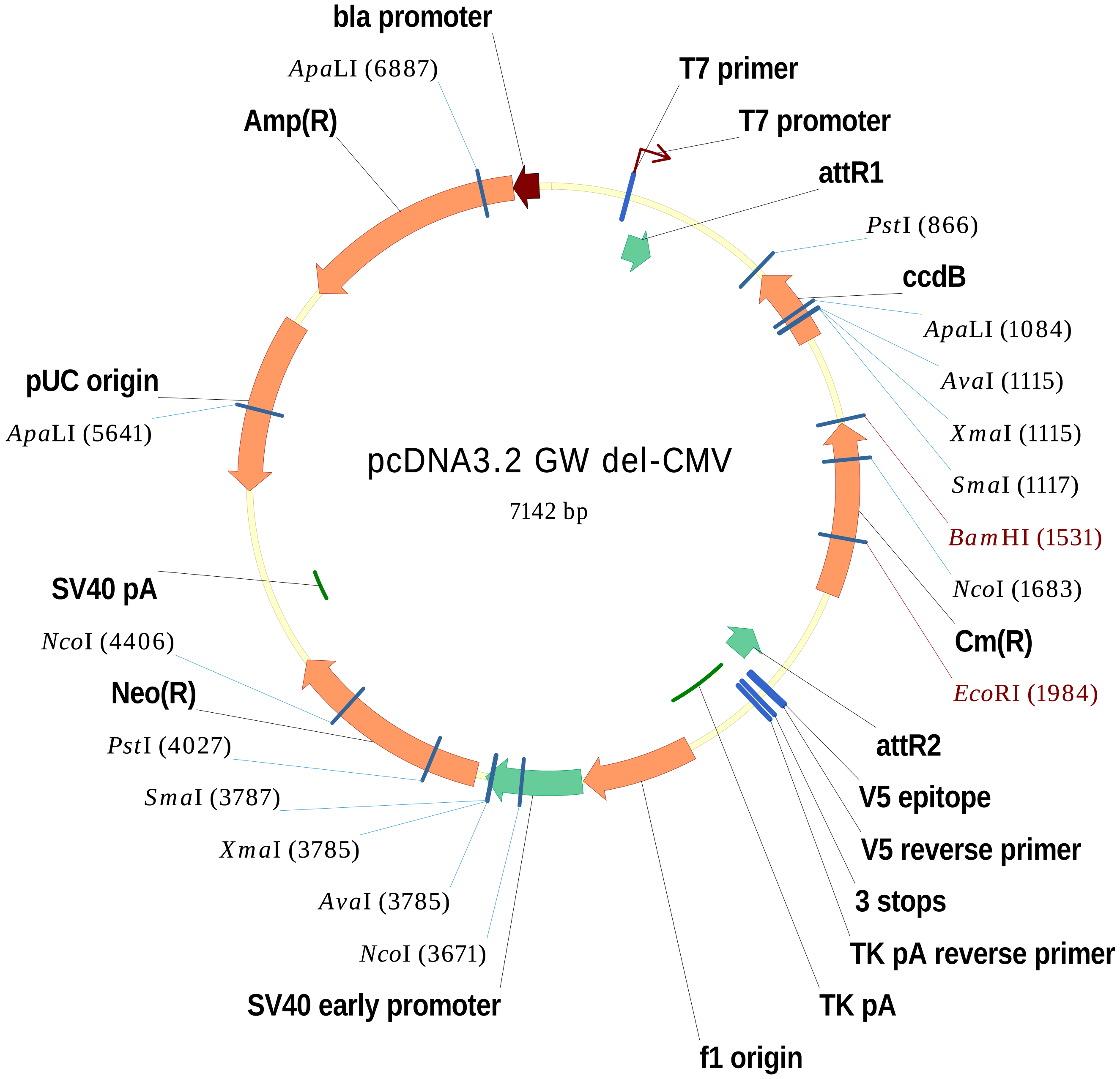

Supplement: Additional file 2 — Figures S1A-H. Maps of plasmids used in this study. S1A pEntL1L3 tTA-2.pdf. S1B pEntL1L3 EF1a-tTA-2.pdf. S1C pEntR3L2 TetO(fl)-2 (insulator).pdf. S1D pEntR3L2 TetO(sh) mCh-Rs1-2.pdf. S1E pR26 R1R2 RexNeo PI-SceI.pdf. S1F Exp-R26(EF1a-tTA TetO-mCh-Rs1).pdf. S1G Exp-pcDNA3.2(EF1a-tTA TetO-mCh-Rs1).pdf. S1H pcDNA3.2GW-delCMV.pdf. [file scrt52-S2.PDF]
