# Supplementary material for: Constitutive Gs activation using a single-construct tetracycline-inducible expression system in embryonic stem cells and mice
Source: Stem Cell Res Ther. 2011 Mar 4;2(2):11. doi: 10.1186/scrt52 (PMC3226282; doi:10.1186/scrt52)
Supplement: Additional file 3 — Table S2. Additional plasmids created as part of this study with brief descriptions and accession numbers. [file scrt52-S3.DOC]

**Table S2: Additional related constructs**

Maps and Addgene deposit numbers are indicated. These vectors do not contain insulator sequences, allowing them to be used to generate viral constructs.

| **Map** | **Plasmid** | **Comments** | **Addgene #** |
| --- | --- | --- | --- |
| **L1L3 Entry Vectors** | | | |
| 2A | pEntL1L3-MCS | Empty entry vector carrying Gateway L1L3 sites, with MCS (full MCS is not shown on map). This plasmid can act as an empty carrier when combined with a R3L2 plasmid to generate expression plasmids carrying just the R3L2 component (i.e., TetO-transgene expressing plasmid). KanR. | 26798 |
| 2B | pEntL1L3 tTA-3 | Gateway L1L3 entry vector containing the tTA regulator. Promoters can be inserted using PacI and SbfI. No insulator sequence. KanR. | 27105 |
| 2C | pEntL1L3 rtTA-3 | Gateway L1L3 entry vector containing the rtTA regulator. Promoters can be inserted using PacI and SbfI. No insulator sequence. KanR. | 27106 |
| 2D | pEntL1L3 EF1a-tTA-3 | Contains Gateway L1L3 sites and EF1α promoter driving tTA. No insulator sequence. KanR | 24415 |
| **R3L2 Entry Vectors** | | | |
| 2E | pEntR3L2-MCS | Empty entry vector carrying Gateway R3L2 sites, with MCS (full MCS is not shown on map). This plasmid can act as an empty carrier when combined with a L1L3 plasmid to generate expression plasmids carrying just the L1L3 component (i.e., a tTA expressing plasmid). KanR. | 26799 |
| 2F | pEntR3L2 TetO(fl)-3 | Contains Gateway R3L2 sites. Full-length (7 repeat) TetO. SbfI, FseI, AvrII, and SalI sites can be used to insert gene of interest, driven by TetO. No insulator sequence. KanR. | 27107 |
