# Supplementary material for: Constitutive Gs activation using a single-construct tetracycline-inducible expression system in embryonic stem cells and mice
Source: Stem Cell Res Ther. 2011 Mar 4;2(2):11. doi: 10.1186/scrt52 (PMC3226282; doi:10.1186/scrt52)

Supplemental Figure S2A

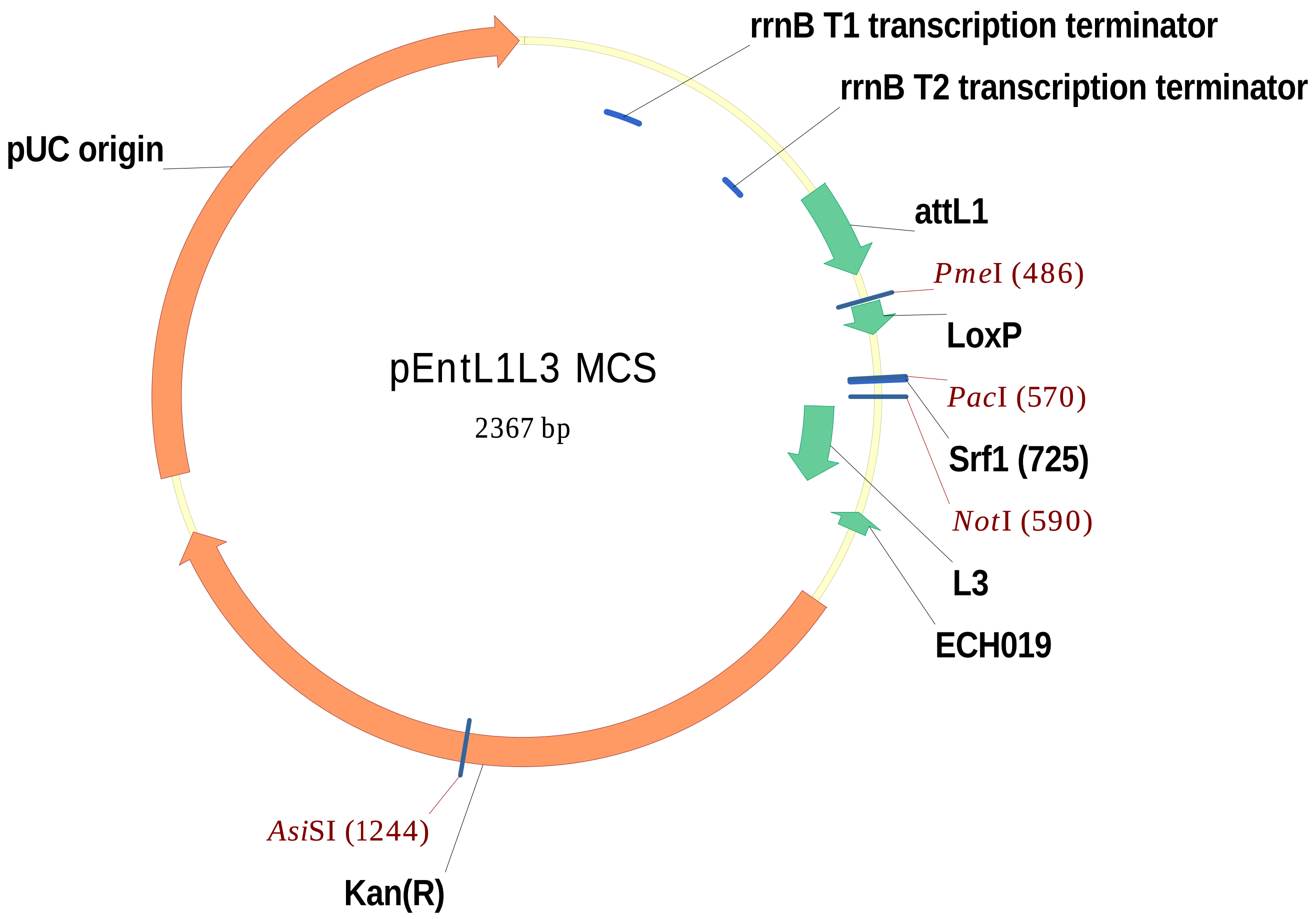

Supplemental Figure S2B pEntL1L3 tTA-3 (no ins)

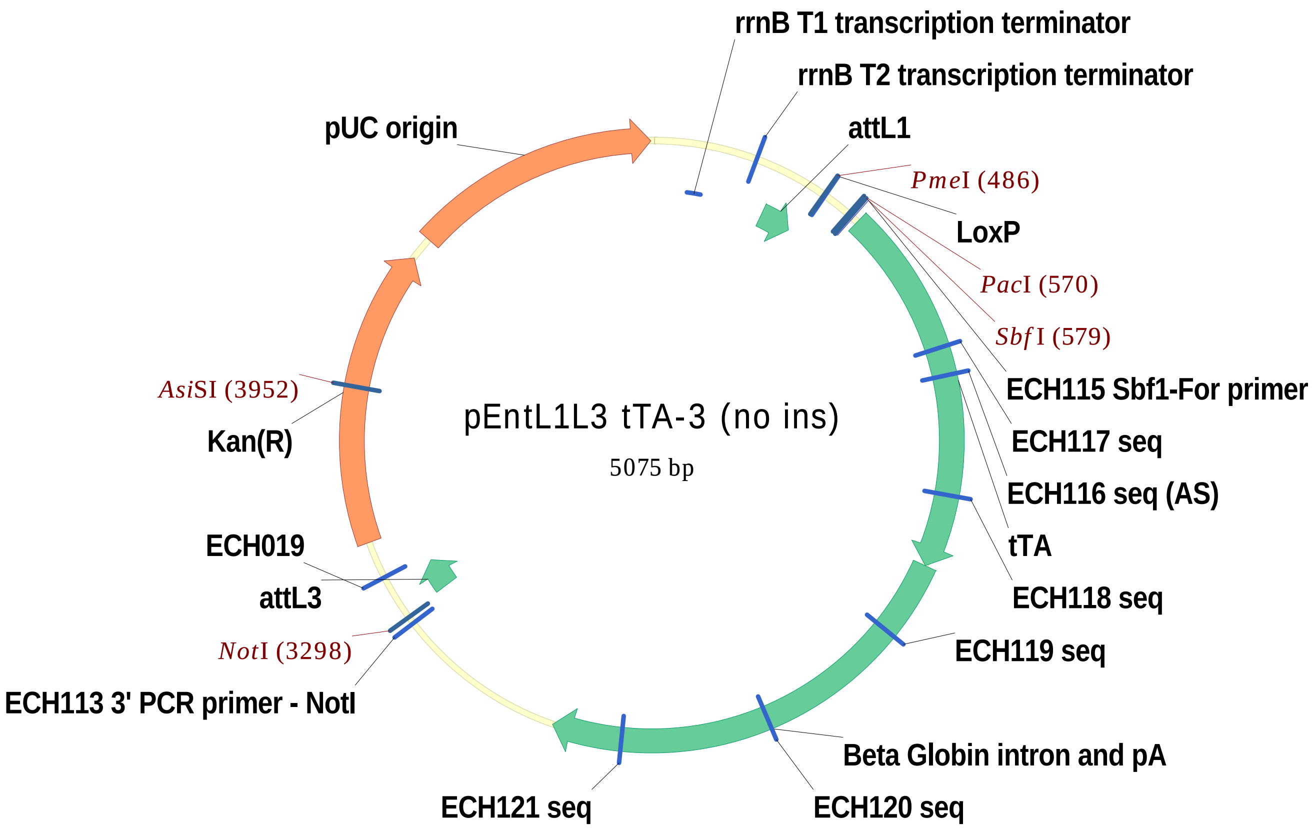

Supplemental Figure S2C pEntL1L3 rtTA-3

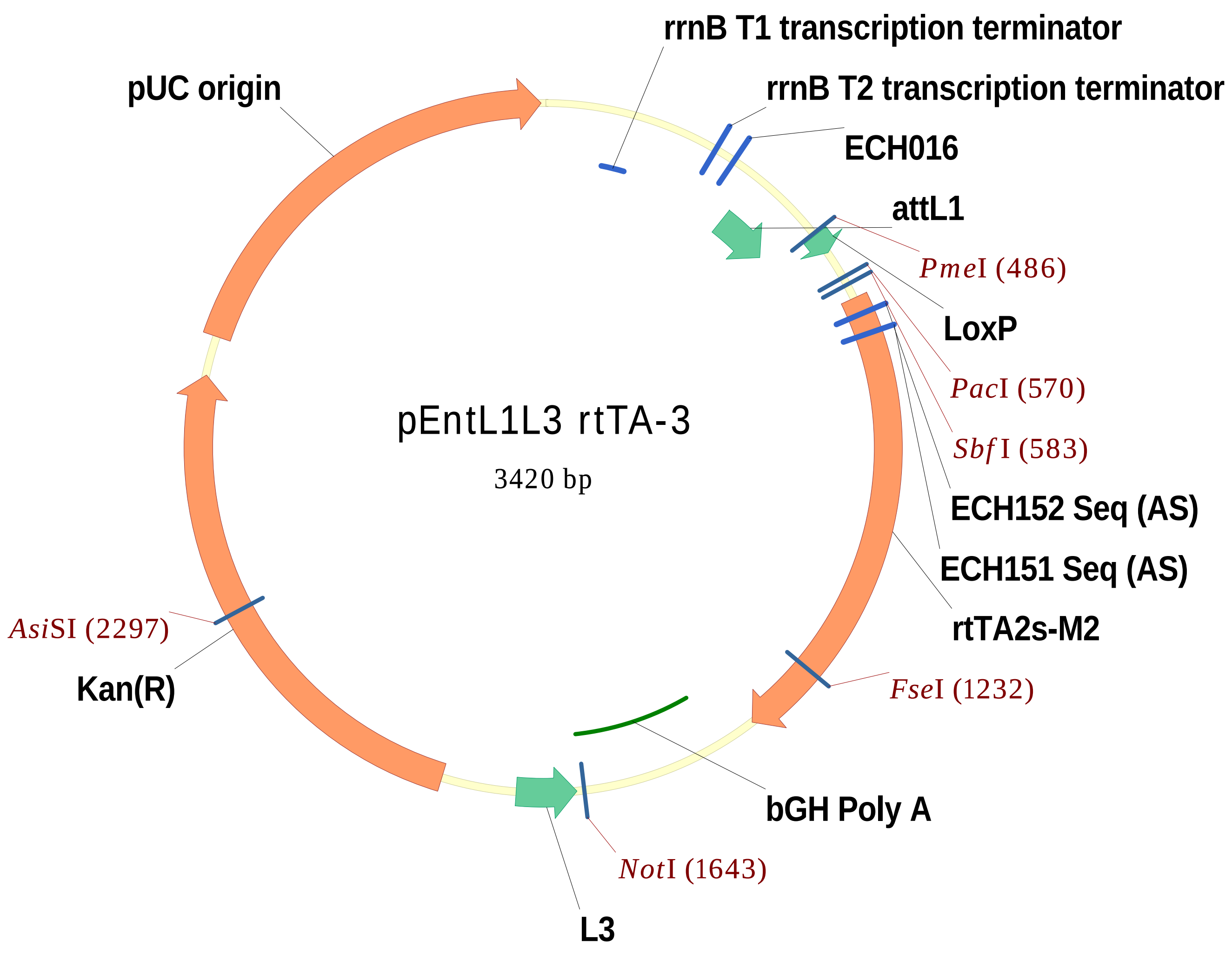

Supplemental Figure S2D

pEntL1L3 EF1a-tTA-3 (no insulator)

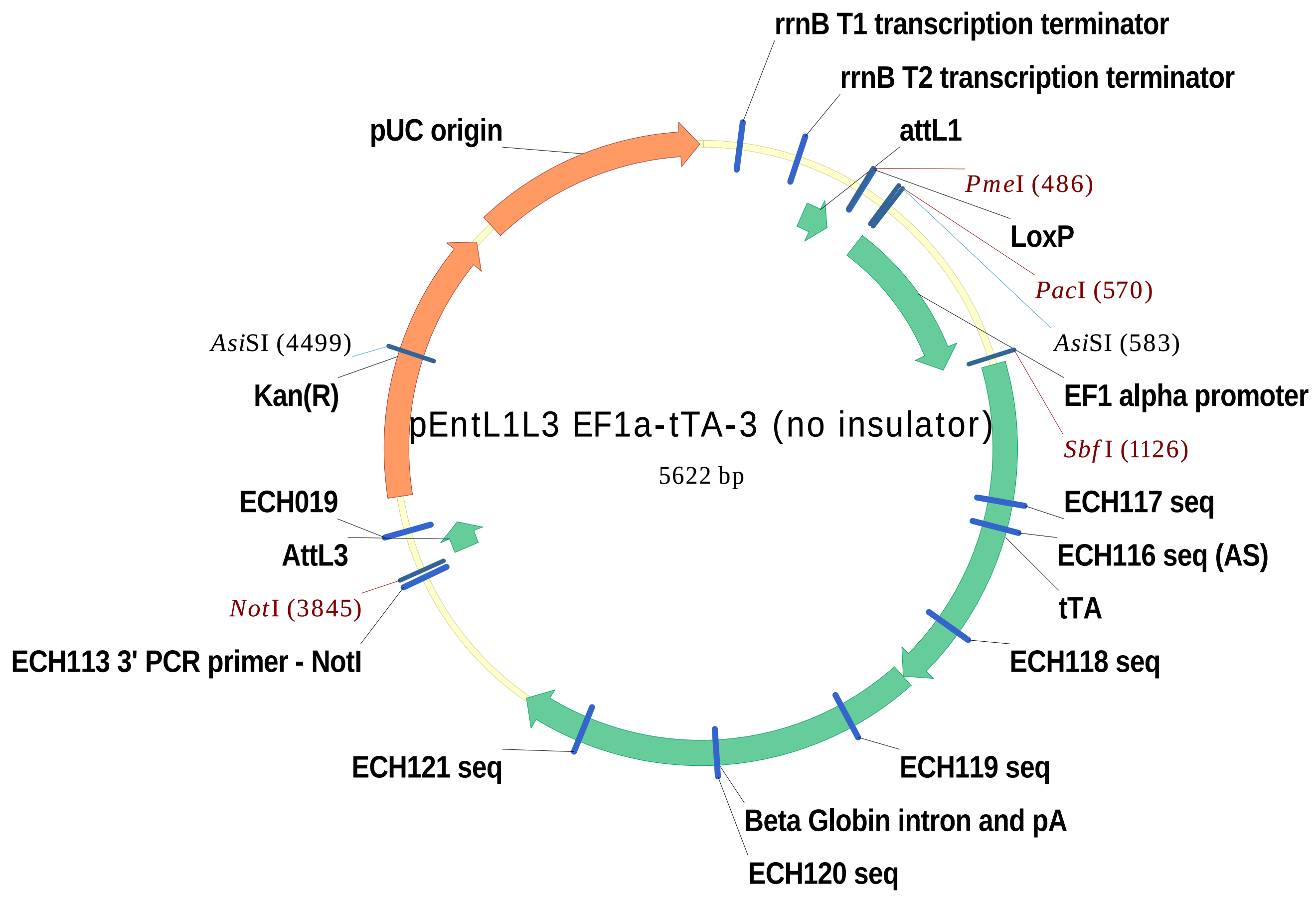

Supplemental Figure S2E

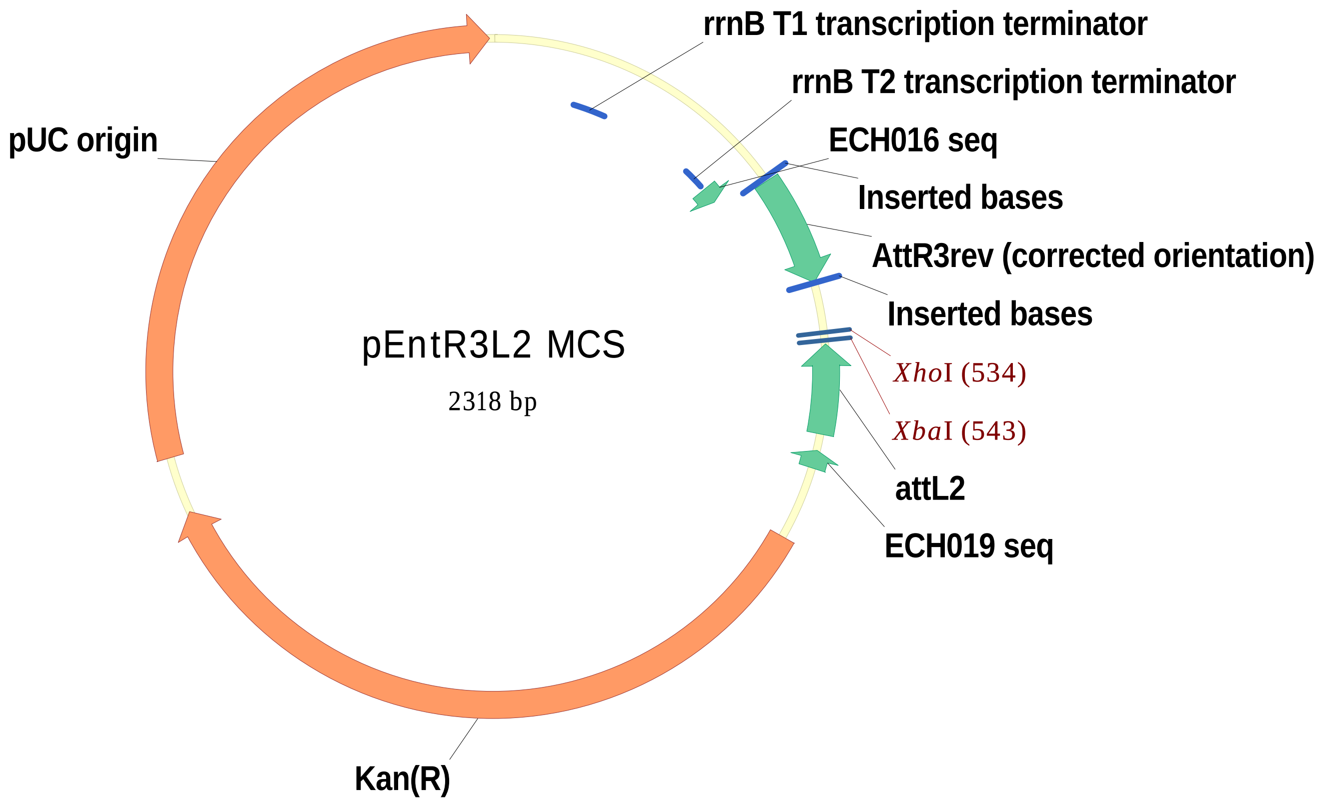

Supplemental Figure S2F

pEntR3L2 TetO(fl)-3 (no insulator)

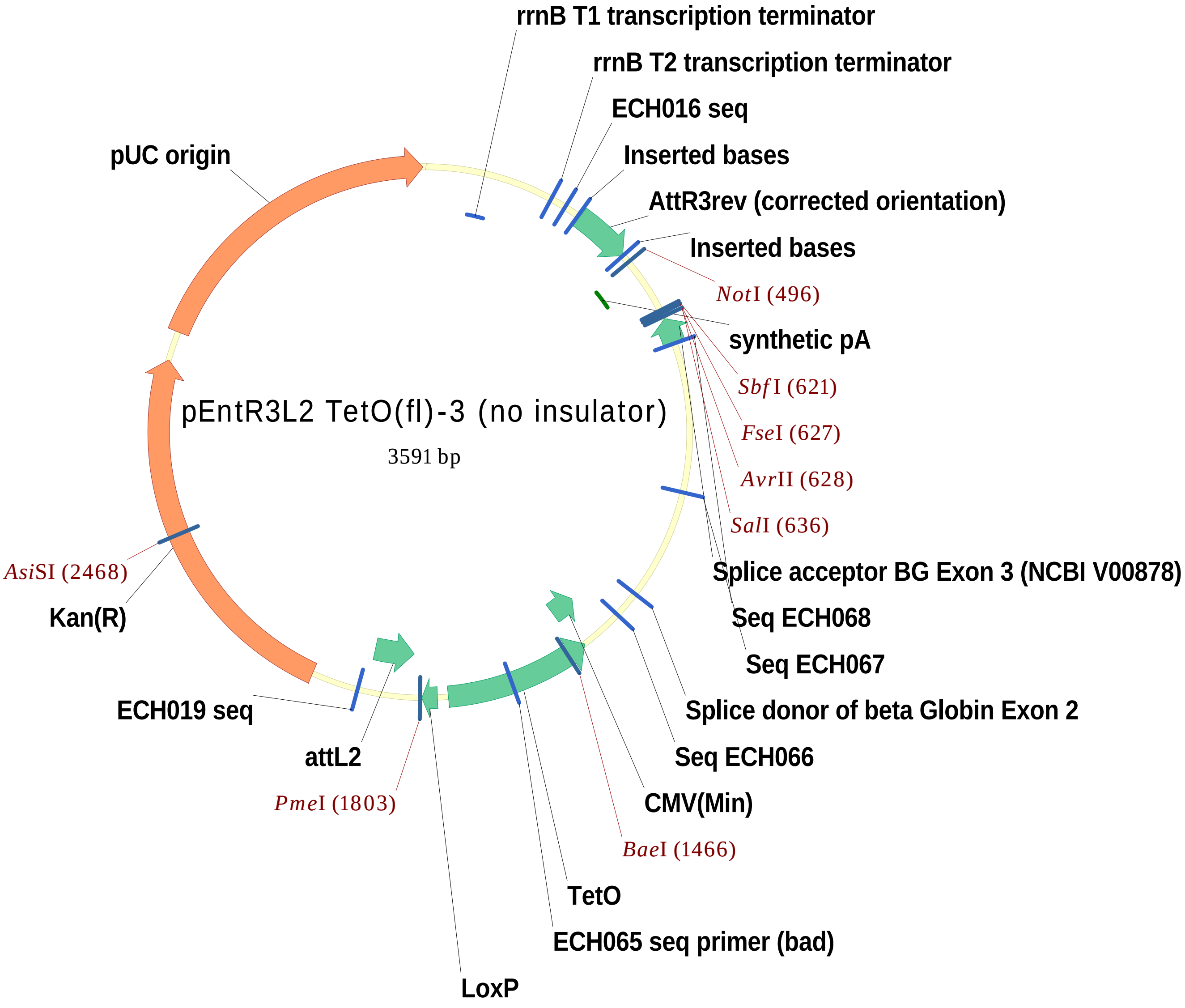

Supplement: Additional file 4 — Figures S2A-F. Maps of additional plasmids described in this study. S2A pEntL1L3 MCS.pdf. S2B pEntL1L3 tTA-3 (no ins).pdf. S2C pEntL1L3 rtTA-3 (no ins).pdf. S2D pEntL1L3 EF1a-tTA-3 (no insulator).pdf. S2E pEntR3L2 MCS.pdf. S2F pEntR3L2 TetO(fl)-3 (no insulator).pdf. [file scrt52-S4.PDF]
